# Optimization of HIV Sequencing Method Using Vela Sentosa Library on Miseq Ilumina Platform

**DOI:** 10.3390/genes15020259

**Published:** 2024-02-19

**Authors:** Nasserdine Papa Mze, Cécile Fernand-Laurent, Solen Daugabel, Olfa Zanzouri, Stéphanie Marque Juillet

**Affiliations:** Service de Biologie, Unité de Microbiologie, Hôpital Mignot, Centre Hospitalier de Versailles, 177 rue de Versailles, 78150 Le Chesnay, Franceozanzouri@ght78sud.fr (O.Z.); smarquejuillet@ght78sud.fr (S.M.J.)

**Keywords:** human immunodeficiency virus, Vela Sentosa, Miseq, personal genome machine

## Abstract

Genotypic testing is often recommended to improve the management of patients infected with human immunodeficiency virus (HIV). To help combat this major pandemic, next-generation sequencing (NGS) techniques are widely used to analyse resistance to antiretroviral drugs. In this study, we used a Vela Sentosa kit (Vela Diagnostics, Kendall, Singapore), which is usually used for the Ion Torrent personal genome machine (PGM) platform, to sequence HIV using the Illumina Miseq platform. After RNA extraction and reverse transcriptase-polymerase chain reaction (RT-PCR), minor modifications were applied to the Vela Sentosa kit to adapt it to the Illumina Miseq platform. Analysis of the results showed the same mutations present in the samples using both sequencing platforms. The total number of reads varied from 185,069 to 752,343 and from 642,162 to 2,074,028 in the Ion Torrent PGM platform and the Illumina Miseq platform, respectively. The average depth was 21,955 and 46,856 for Ion Torrent PGM and Illumina Miseq platforms, respectively. The cost of sequencing a run of eight samples was quite similar between the two platforms (about USD 1790 for Illumina Miseq and about USD 1833 for Ion Torrent PGM platform). We have shown for the first time that it is possible to adapt and use the Vela Sentosa kit for the Illumina Miseq platform to obtain high-quality results with a similar cost.

## 1. Introduction

Human immunodeficiency virus (HIV) infection remains a major public health problem. Human immunodeficiency virus/acquired immunodeficiency syndrome (HIV/AIDS) has caused the deaths of 40.4 million people since the start of the epidemic in the 1980s [1]. According to the latest available data, in 2022, 630,000 people died of HIV/AIDS-related illnesses, and 1.3 million people contracted HIV worldwide [1]. In France, 6.5 million HIV serology tests were performed by medical diagnostic laboratories in 2022 [2]. According to the estimates made by the French Ministry of Health, between 4200 and 5700 people were tested positive for HIV in 2022. This number has remained stable for several years, regardless of the mode of contamination or place of birth of those who were diagnosed to be HIV-positive. In 2022, 28% of HIV infections were discovered at an advanced stage of infection in France, and this proportion has remained stable over the past few years. Among the patients whose HIV status was confirmed in 2022, 54% were heterosexuals, 41% were men who had sex with other men (MSM), 2% were transsexuals that contracted HIV through sexual intercourse, and 1% was intravenous drug users (IDUs). Less than 1% were children under 15 years old, a large majority of whom were infected through mother-to-child transmission. Also, in 2022, 43% of HIV infections were diagnosed for the first time at a late stage. At the regional level, the incidence of HIV infection remained markedly high in the French overseas regions, hence, the importance of maintaining a high level of screening activity in those regions [2].

Currently, 23.3 million people worldwide are on anti-HIV treatment, the efficacy of which has considerably improved over the years. Today, a person living with HIV who starts an antiretroviral (ARV) treatment would have the same life expectancy as an HIV-negative person of the same age. ARV treatment prevents HIV-related illness and disability and works best when started very early in the course of HIV infection. The World Health Organization (WHO) recommends two nucleoside reverse transcriptase inhibitors (NRTIs) plus a non-nucleoside reverse transcriptase inhibitor or an integrase inhibitor for the standard first-line ARV therapy in adults and adolescents [3]. In adults, the second-line ARV therapy consists of two NRTIs combined with a ritonavir-boosted protease inhibitor. NRTIs are molecules that are active against HIV that block the enzyme that enables the synthesis of complementary DNA (cDNA) from viral RNA, prior to its integration into the genome of the infected cell. These inhibitors compete with natural nucleosides, preventing DNA chain elongation [4,5]. Non-nucleoside reverse transcriptase inhibitors (NNRTIs) have a direct inhibitory effect on HIV-1 reverse transcriptase by forming a reversible, non-competitive bond with the enzyme. NNRTIs act as non-competitive antagonists by binding to a hydrophobic region adjacent to the catalytic site of reverse transcriptase (RT) [6,7]. Protease inhibitors are molecules that bind to the active site of HIV protease [8], resulting in the formation of immature, non-infectious viral particles. HIV integrase inhibitors are molecules active against HIV-1 and HIV-2, targeting HIV integrase, the enzyme required to integrate the HIV genome into the DNA of the host cell. The inhibition of integrase prevents the integration of HIV genome into the host cell DNA, thus blocking retrovirus formation and the spread of viral infection.

ARV treatment works best when taken as prescribed by medical practitioners. Poor compliance can lead to drug resistance, enabling HIV to multiply and the disease to progress. In addition to poor compliance, another major obstacle to a successful ARV therapy is the development and acquisition of drug resistance due to mutations in HIV strains. Genotypic tests for ARV resistance are widely used to improve the therapeutic management of patients [9,10]. They are recommended at the time of diagnosis, i.e., before the initiation of treatment, in order to search for the possible transmission of resistance, which is detected in around 10% of new infections for non-NRTIs and 3% for anti-proteases [9]. Further genotypic tests are strongly recommended in the event of ARV treatment failure. Genotypic tests analyse the sequences of genes that code for viral reverse transcriptase, protease, and integrase, in comparison with those of a wild-type HIV reference strain, to identify the mutations associated with resistance [11,12,13,14]. ARV resistance is a result of the accumulation of specific mutations in HIV genes, and the presence of these mutations significantly reduces the efficacy of ARV and prolongs the duration of ARV therapy to attain curative effects [15,16,17]. The genotypic analysis of HIV isolates can detect the presence of mutations that are associated with phenotypic or clinical resistance to different ARVs [18]. There is generally a good correlation between the number of mutations associated with resistance to different drugs and the increase in the 50% inhibitory concentration (IC_50_).

At the Versailles Hospital Centre in Versailles, France, personal genomic machine (PGM) Ion Torrent high-throughput sequencing technology has been used, together with a commercial kit from Vela Diagnostics, since 2021, to diagnose HIV. Compared with the older diagnostic techniques, such as the Sanger dideoxy method, next-generation sequencing (NGS) enables the simultaneous reads of millions of copies of HIV genomes per run, enabling the detection of low-frequency mutations. In addition, NGS approaches enable the detection of admixtures, which is particularly important for patient follow-up [19]. In 2019, Vela Diagnostics NGS obtained the United States Food and Drug Administration (FDA) approval for HIV genotyping and resistance testing for in vitro diagnostic purposes [20].

A recent Italian study that evaluated the Vela Diagnostics system showed better results with this NGS technology, compared to Sanger sequencing, in terms of the success rate, accuracy, repeatability, and reproducibility of results [21]. Several other studies conducted prior to this Italian study had evaluated and demonstrated the performance of the Vela Dx Sentosa next-generation sequencing system for HIV-1 cDNA genotypic resistance [22,23,24,25]. In the present study, for the first time, we used a Vela Sentosa kit to sequence HIV genomes using the Illumina Miseq platform. The aim of our study was to show that it is possible to adapt the Vela kit to Miseq and obtain better results at a lower cost than with the PGM Ion Torrent sequencer.

## 2. Materials and Methods

### 2.1. Test Validation Samples

The reference HIV samples for quality control (QC) and test validation were obtained from the French National Reference Centre for HIV (Centre National de Référence HIV; CNR) in Bichat hospital, Paris, France. These samples are produced from the supernatants of the reference HIV strains under in vitro cultivation. Each year, QC samples are distributed and tested in several laboratories in France and at the Pasteur Institute (Paris, France) to assess the quality of HIV tests being performed. In addition to the QCs, we added a positive HIV control (AcroMetrix^TM^ Genotyping HIV RT/PR mutant control, AcroMetrix, Benicia, CA, USA) and a negative control (HIV-free PCR).

### 2.2. RNA Extraction

All samples were centrifuged at 17,000× *g* for 2 h at 4 °C. After centrifugation, 400 µL of the supernatant was used to extract the viral RNA using the EZ1 virus mini kit (Qiagen S.A.S., Courtaboeuf, France) as recommended by the manufacturer.

### 2.3. PCR Amplification

Reverse transcriptase-polymerase chain reaction (RT-PCR) was performed using the Sentosa^®^ SQ HIV drug resistance mutations (DRM) library kit (Vela Diagnostics, Hamburg, Germany), according to the manufacturer’s recommendations. Four primer pools were used to amplify the protease (PR), RT, and integrase (INT) genes. RT-PCR was performed with a single thermal cycling program using 1.25 µL of RT enzyme mix, 1.25 µL of each primer pool, and 10 µL of eluted RNA. The RT program was 25 °C for 2 min and 50 °C for 15 min. The PCR amplification conditions were as follows: initial denaturation at 94 °C for 2 min, followed by 50 cycles consisting of 94 °C for 15 s, 58 °C for 15 s, 57 °C for 15 s, 56 °C for 15 s, 55 °C for 15 s, 54 °C for 15 s, 53 °C for 15 s, and 68 °C for 2 min, and followed by the final extension step at 68 °C for 1 min. Each sample was tested in quadruplicate according to the primer pool used in a single PCR program.

### 2.4. Library Preparation for Ion Torrent PGM Platform

The library was prepared according to the Sentosa^®^ SQ HIV DRM (Experienced User Card V1.0) manual protocol (Vela Diagnostics, Kendall, Singapore). After amplification, the PCR products were normalized and purified using the Sentosa^®^ SQ virus solution prep kit. For each sample, 13 µL of the purified PCR product were used for enzymatic fragmentation using the Sentosa^®^ SQ HIV DRM library kit. The ligation step was performed using 1 µL adapter and 1 µL barcode in the Ion Xpress Barcode Adapters kit (Thermo Fisher Scientific, Paris, France). The ligation program was 20 °C for 15 min, 65 °C for 5 min, and 85 °C for 2 min. After ligation, each ligated PCR product was purified with magnetic beads and eluted with 25 µL elution buffer. For each sample, 10 µL was taken to constitute a pool of all the libraries. The pool was then diluted by 1/4 with the elution buffer, and PCR and emulsion enrichment were performed on the Ion Chef instrument prior to sequencing. The three viral genes, PR, RT, and INT, were sequenced on a Sentosa^®^ SQ 316 chip (Ion Torrent PGM) using the dedicated Sentosa^®^ reagent kits (Vela Diagnostics, Kendall, Singapore).

### 2.5. Library Preparation for Illumina Miseq Platform

After PCR, 20 µL of the purified product were used for enzymatic fragmentation using the same Sentosa^®^ SQ HIV DRM manual protocol. Ligation was performed using 10 µL of TruSeq DNA UD Indexes v2 from the Illumina platform (Illumina Inc., San Diego, CA, USA) under the following conditions: 20 °C for 20 min, 65 °C for 5 min, and 85 °C for 2 min. After ligation, each ligated PCR product was eluted with 18 µL elution buffer. For each sample, 5 µL was taken to form a pool of all the libraries. The concentration of the pooled library was set at 20 nM, and no dilution was performed. The pool was denatured with 0.05 N NaOH and diluted with HT1 to 30 pM. This diluted pool was incubated at 96 °C for 4 min using a heat block, and 15% PhiX control was added. The library products were analysed by sequencing using the Miseq v2 reagent kit (cycle 300) (Illumina Inc.) on the Illumina Miseq platform in 2 × 151 cycles and a micro flow cell.

### 2.6. Sequence Data Analysis

The bioinformatic analyses were performed using the Advanced Sequencing Platform (ASP) v3.13.0 available from SmartGene (SmartGene, Lausanne, Switzerland). The SmartGene HIV-1 application is based on the proprietary IDNS (Integrated Database Network System) technology of SmartGene and is accessible via a secure web interface, which can handle base called sequencing files generated through different sequencing technologies. Related SmartGene patents are EP05700367 and EP07816282. *.bam or *.fastq files obtained through the Sentosa SQ platform (Vela) or through the MiSeq platform (Illumina) were uploaded to ASP. The application first performs the following steps in an automated manner:

(1) The paired-end detection of read files, if uploaded in the same batch;
(2) Technology-specific quality filtering to trim or remove low-quality reads;
(3) The establishment of a work list of the batch.

The user then selects the analysis pipeline to be used, in this case the HIV-1 PR+RT+INT targeted workflow, and selects the appropriate cut-off for ambiguous bases (background noise filtering, here, 0.5%). The automated analysis pipeline performs the following actions:

(1) Quality filtering: R1 and R2 files are merged for Illumina data, then a sliding window (size of 25 nt) is applied to reads, enhancing the trimming of poor-quality sections having a low Phred score (<23 for the present study) and filtering short reads as well (<20 nt in this study);
(2) Read mapping: reads passing the quality filters are mapped against HIV-1 profiles for PR, RT, and INT, generating a frame-aware nucleic acid alignment, using a proprietary alignment method of combined global and local alignments developed by SmartGene, followed by the translation of the corrected alignments into amino acid sequences;
(3) Mapped reads are used for the creation of a frequency matrix;
(4) Mutations are determined and validated with respect to the reference (here, HxB2, accession no. K03455) and their frequencies are calculated.

To generate the final report, the user selects the mutation cut-off (0.5–20%) above which mutations are assessed for resistance using one of the embedded HIV resistance algorithms, as well as a minimal read depth. In the present study, the algorithm developed by the French National Agency for AIDS Research (ANRS) was used [26], with a cut-off value of 20% and a minimum depth of 200. The drug resistance profile was then characterised, and the mutation frequencies were expressed as a percentage of the total number of HIV strains analysed. The final results were summarized in a list of all mutations per sample with their respective frequencies.

### 2.7. Statistical Analysis

All statistical tests were carried out using Microsoft Excel version 16 (Microsoft Corp., Redmond, WA, USA). The cost per sample per run (eight samples) was calculated for the sequencing preparation workflow of Illumina Miseq and Ion Torrent PGM platforms. The prices of all kits used from PCR to sequencing were included, taking into account the total number of samples that can be processed per kit.

The average values of depths and the numbers of reads were compared using the two-tailed Student *t*-test. The significance level was set at *p* < 0.05.

Cohen’s kappa coefficient was calculated to estimate the degree of agreement between the results of Ion Torrent PGM platform and Miseq Illumina platform [27].

## 3. Results

**Figure 1:**
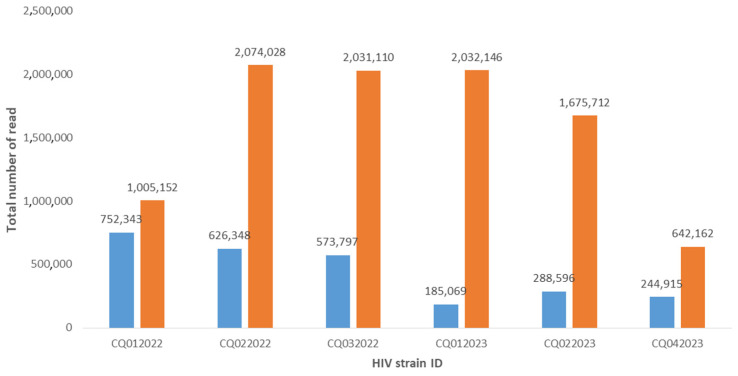
Comparison of the total number of reads with Ion Torrent PGM (blue bars) and Illumina Miseq (orange bars) platforms.

**Figure 2:**
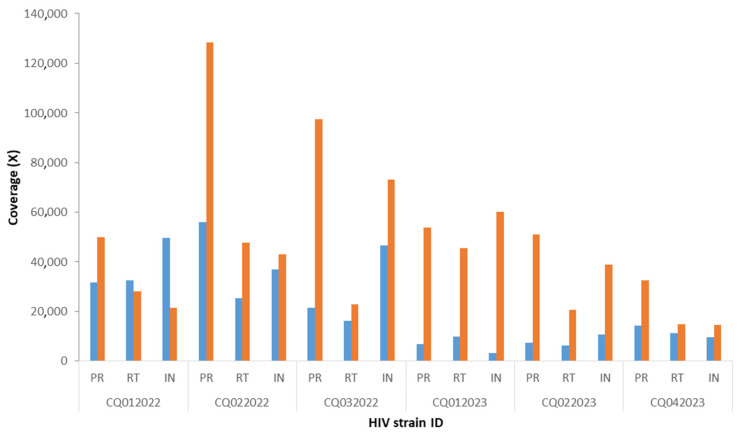
Comparison of depth between Ion Torrent PGM and Illumina Miseq platforms. The figure presents the average coverage for each HIV gene with PGM Ion Torrent (blue squares) and Miseq Illumina (orange squares). PR, protease gene; RT, reverse transcriptase gene; and INT, integrase gene. “CQ” followed by a number refers to different HIV strains.

A total of six QCs and the single positive control were correctly sequenced, and all mutations were found with both platforms. For the Ion Torrent PGM platform, the total number of reads ranged from 185,069 to 752,343 (Figure 1). The average depth was 21,955, with an average mutation frequency of 99.3% (Figure 2). All HIV strains showed mutations that are associated with drug resistance. CQ022022, CQ032022, CQ012023, and CQ042023 strains belonged to the genetically distinct group referred to as subtype B, while CQ022023 strain was characterized as subtype A, and CQ012022 strain had genetic features of subtype F2 (Supplementary File S1).

For the Miseq Illumina platform, the total number of reads ranged from 642,162 to 2,074,028. The average depth was 46,856, with an average frequency of 99.5%. CQ022022, CQ032022, CQ012023, and CQ042023 strains were characterized as HIV-1 subtype B, while CQ022023 strain was determined to be subtype A1, and CQ012022 strain belonged to subtype F2 (Supplementary File S2).

Concerning the number of mutations, those found in the INT gene were 100% concordant (Cohen’s kappa coefficient: 1; perfect agreement) for all samples. However, a few small, non-significant discrepancies (*p* > 0.05) were observed in the PR and RT genes (Table 1). These discordant nucleotides have not been reported to be associated with drug resistance.

The comparison of mean reads showed a statistically significant difference between Miseq (mean total: 577,718) and PGM (mean total: 445,178) (*p* = 0.0017). Although the average depth was higher with the Illumina Miseq platform than with the Ion Torrent PGM platform, the difference did not reach statistical significance (*p* = 0.10).

**Figure 3:**
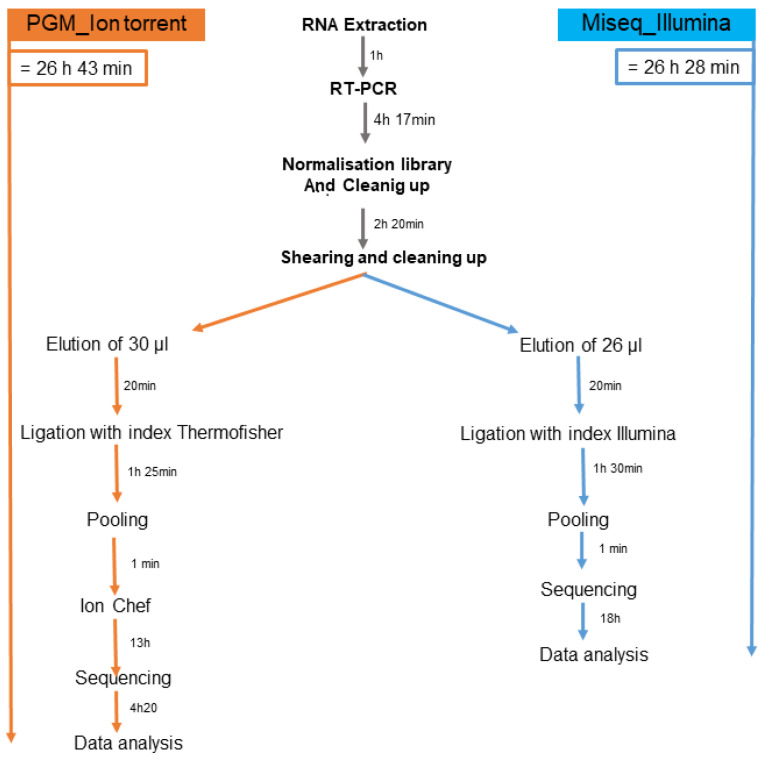
Sequencing times with Illumina Miseq and Ion Torrent PGM.

Furthermore, the total duration of the preparation run was 26 h 43 min with the PGM and 26 h 28 min with Miseq. These are times taken from real runs of the protocol. In all cases, the handling of the Illumina protocol was simpler and shorter (Figure 3).

The price of a sequencing run on the Illumina Miseq platform was EUR 1640 or about USD 1790 (exchange rate on 5 January 2024), and EUR 1685 or about USD 1833 on the Ion Torrent PGM platform (Table 2).

## 4. Discussion

The NGS technology has become a powerful tool in the public health context of virus surveillance. The study of HIV drug resistance by sequencing is a key step in HIV/AIDS therapeutic management and strategy. Sequencing performed in the search for mutations in RT, PR, and INT genes is indicated in several clinical situations to optimise patient management [11,12,13,14].

The most common NRTI resistance-associated mutations are M184V/I. Other common discriminating mutations include K65R, K70E/G/Q, L74V/I, Y115F, and the Q151M mutation complex. The most common NNRTI resistance-associated mutations are L100I, K101E/P, K103N/S, V106A/M, Y181C/I/V, Y188C/H/L, G190A/S/E, and M230L. Each of these is associated with an intermediate-to-high level of phenotypic resistance to nevirapine. The mutations associated with PI resistance are D30N and N88D, which are major mutations associated with resistance to nelfinavir. In addition, L10F, V11I, K20TV, L23I, K43T, F53L, Q58E, A71IL, G73STCA, T74P, N83D, and L89V are known to be common non-polymorphic accessory mutations that may occur in PI resistance [28].

In France, the prevalence of viruses carrying at least one mutation associated with ARV resistance was 9.2% in 2014 [29]. This prevalence rate of ARV-resistant viruses has remained stable over time, i.e., between 10 and 13%. In comparison, during the same period in France, the prevalence rates of viruses carrying mutations associated with resistance to NRTIs, the first-generation NNRTIs, or PIs were 4.3%, 3%, and 2.4%, respectively. The prevalence of mutant HIV strains with mutations associated with resistance to rilpivirine and/or etravirine was 6%, with the majority of mutations occurring at position 138. Resistance to at least one NNRTI was found in 8.4% of HIV isolates. Mutations associated with resistance to INT inhibitors were observed in 2.7% of cases (four clinical isolates with E157Q mutation and two isolates with R263K mutation that confer resistance to dolutegravir). Moreover, the frequency of viral resistance is similar in patients infected with non-B subtypes than in patients infected with B subtype HIV-1 [30].

Since the introduction of PGM Ion Torrent sequencing technology at the Hospital Centre in Versailles in 2021, this technique has been used to study HIV resistance in over 677 patients. Ion Torrent sequencing technology is known for its simplicity, speed, and lower cost, due to its semiconductor chips [31].

The switch to a new sequencer, i.e., the Illumina Miseq platform, at our laboratory prompted us to make adjustments and adapt the Sentosa^®^ kit supplied by Vela to the Miseq sequencer on the Illumina platform. Vela’s Sentosa kit is usually used for the PGM; this is the first time that it has been adapted for use with the Miseq Illumina. The advantage of using the Vela kit is that a single PCR can amplify all three HIV genes of interest, namely, RT, PR, and INT [32], when compared with other techniques that require PCR amplifications of individual genes [33].

Overall, the results showed a perfect concordance (Cohen’s kappa coefficient ranged from 0.97 to 1; perfect agreement) of mutations between the two techniques, validating the Miseq sequencing technique on the Illumina platform. Indeed, the latter assay performed with minor modifications met our predefined validation criteria based on recommended methods for validating an in-house genotyping assay for HIV drug resistance monitoring (Figure 3). Consequently, the test was deemed suitable for our needs and capable of replacing the Ion Torrent PGM test in our laboratory.

Interestingly, the total number of reads for each sample and the average depth were higher with the Miseq Illumina platform than with the Ion Torrent platform. This difference can be explained by the fact that the chip in Ion Torrent platform used for sequencing generates around 5 million reads, whereas Illumina platform’s micro flow cell generates around 8 million reads. In addition, the preparation of the library was simpler and faster with Miseq Illumina platform, allowing time flexibility for laboratory technicians and the rapid delivery of results. Moreover, the price of reagents was USD 1790 with the Miseq Illumina platform and USD 1833 with the Ion Torrent PGM platform. This small difference in price is due to the necessity of using several Thermo Fisher Scientific kits for library enrichment on the Ion Chef automated system prior to PGM sequencing. This enrichment step is specific to and necessary for the Ion Torrent platform, but not for the Miseq Illumina platform.

## 5. Conclusions

For the first time, we have shown that it is possible to use the Vela library to sequence HIV genome on the Miseq sequencer with Illumina platform. This platform is simpler and faster and provides high-quality results similar to those of the Ion Torrent PGM platform.

## Figures and Tables

**Table 1:** Agreement and discordance in the number of mutations (>20%).

Gene	ID HIV Strain Number	Mutation Number PGM > 20%	Mutation Number Miseq > 20%	Number of Discordance	Resistance Yes or No	Concordance (%)
Protease	CQ012022	24	23	1	no	99
	CQ022022	18	18	0	yes	100
	CQ032022	19	20	1	no	99
	CQ012023	24	24	0	yes	100
	CQ022023	11	11	0	yes	100
	CQ042023	14	14	0	yes	100
Reverse Transcriptase	CQ012022	43	43	0	yes	100
	CQ022022	38	37	1	no	99.5
	CQ032022	22	23	1	yes	99.5
	CQ012023	31	31	0	yes	100
	CQ022023	34	34	0	yes	100
	CQ042023	26	25	1	no	99.5
Integrase	CQ012022	20	20	0	yes	100
	CQ022022	10	10	0	yes	100
	CQ032022	18	18	0	yes	100
	CQ012023	22	22	0	yes	100
	CQ022023	21	21	0	yes	100
	CQ042023	15	15	0	yes	100

**Table 2:** Price comparison between Ion Torrent PGM and Illumina Miseq platform.

Library Reagent	Manufacturer	Price (EUR)	Price, 1 run (EUR)	Total Price	
				PGM_Ion Torrent	Miseq_Illumina
Sentosa SQ HIV DRM Library Kit (96 samples)	Vela	2644	220.33	1685.05	1640.24
Sentosa SQ Virus Solution Prep Kit (96 samples)	Vela	974.05	81.17		
MiseqR Reagent kit v2 (300 cycles)	Illumina	1273	1273		
IDT for Illumina—TruSeq DNA UD Indexes v2 (96 samples)	Illumina	788.88	65.74		
Ion Xpress Barcode Adapters Kit (96 samples)	ThermoFisher	1757.97	146.49		
Ion PGM^TM^ Hi-Q^TM^ View Chef Reagent (four reactions)	ThermoFisher	3171.92	792.98		
Ion PGM^TM^ Hi-Q^TM^ View Chef Supplies (four reactions)	ThermoFisher				
Ion PGMTM Hi-QTM View Chef Solutions (four reactions)	ThermoFisher				
Ion PGMTM Hi-QTM View Sequencing Supplies (four reactions)	ThermoFisher				
Ion PGM Hi-Q View Sequencing Solutions (four reactions)	ThermoFisher				
Ion PGM Hi-Q View Sequencing dNTPs (four reactions)	ThermoFisher				
Sentosa SQ 318 Chip Kit (one reaction)	Vela	3552.6	444.07		

## Data Availability

No new data were created or analyzed in this study. Data sharing is not applicable to this article.

## Supplementary Materials

The following supporting information can be downloaded at: https://www.mdpi.com/article/10.3390/genes15020259/s1, Supplementary File S1: Reports of drug resistance results with the Ion Torrent PGM platform; Supplementary File S2: Reports of drug resistance results with the Illumina Miseq platform; Supplementary File S3: Frequency of mutations with their depths found in all samples with both platforms.

## Abbreviations

AIDS, acquired immunodeficiency syndrome; ANRS, French National Agency for AIDS Research; ARV, antiretroviral; ASP, Advanced Sequencing Platform; cDNA, complementary DNA; CNR, Centre National de Référence (French National Reference Centre for HIV); DRM, drug resistance mutations; FDA, Food and Drug Administration; HIV, human immunodeficiency virus; IC_50_, 50% inhibitory concentration; IDNS, Integrated Database Network System; IDU, intravenous drug users; INT, integrase; MSM, men who had sex with other men; NGS, next-generation sequencing; NRTIs, nucleoside reverse transcriptase inhibitors; NNRTI, non-nucleoside reverse transcriptase inhibitors; PGM, personal genomic machine; PR, protease; QC, quality control; RT, reverse transcriptase; RT-PCR, reverse transcriptase-polymerase chain reaction; USD, United States dollars; and WHO, World Health Organization.
