# Supplementary material for: Optimization of HIV Sequencing Method Using Vela Sentosa Library on Miseq Ilumina Platform
Source: Genes (Basel). 2024 Feb 19;15(2):259. doi: 10.3390/genes15020259 (PMC10887851; doi:10.3390/genes15020259)
Supplement: Supplementary file 1 [file genes-15-00259-s001.zip › Supplementary material/File S1.pdf]

SmartGene

NGS - Drug Resistance Report

Sample ID: CQ012022_PGM **Source ID:** CQ012022_PGM **MID:** n.a.

Subtype: F2

Drug resistances

Drug resistance algorithm: ANRS (2022.33/sg/b)

Nucleoside Reverse Transcriptase Inhibitors (NRTI)

Drug	Mutations list	Range	Color	Interpretation
3TC_FTC		1		S - Susceptible
ABC	**41L (100.0%)**, **67N (98.4%)**, **74V (99.2%)**, **215F (98.4%)**	3		R - Resistance
ISL		1		S - Susceptible
TDF_TAF	**41L (100.0%)**, **67N (98.4%)**, **69D (98.8%)**, **74V (99.2%)**, **215F (98.4%)**	3		R - Resistance
ZDV	**41L (100.0%)**, **67N (98.4%)**, **70R (99.1%)**, **215F (98.4%)**, **219Q (95.6%)**	3		R - Resistance

Non-Nucleoside Reverse Transcriptase Inhibitors (NNRTI)

Drug	Mutations list	Range	Color	Interpretation
DOR	**181C (100.0%)**, **190A (100.0%)**	2		I - Possible resistance
EFV	**181C (100.0%)**, **190A (100.0%)**	3		R - Resistance
ETR	**181C (100.0%)**, **190A (100.0%)**	3		R - Resistance
NVP	**181C (100.0%)**, **190A (100.0%)**	3		R - Resistance
RPV	**181C (100.0%)**	3		R - Resistance

Protease Inhibitors (PI)

Drug	Mutations list	Range	Color	Interpretation
ATV_RTV	**10V (100.0%)**, **16E (99.7%)**, **33F (100.0%)**, **90M (100.0%)**	3		R - Resistance
DRV_RTV_BID	**33F (100.0%)**	1		S - Susceptible
DRV_RTV_QD	**33F (100.0%)**	1		S - Susceptible
LPVr	**10V (100.0%)**, **33F (100.0%)**, **53L (100.0%)**, **54V (100.0%)**, **82F (100.0%)**, **90M (100.0%)**	3		R - Resistance

Integrase Strand Transfer Inhibitors (INSTI)

Drug	Mutations list	Range	Color	Interpretation
BIC		1		S - Susceptible
CAB		1		S - Susceptible
DTG_BID		1		S - Susceptible
DTG_QD		1		S - Susceptible
EVG		1		S - Susceptible
RAL		1		S - Susceptible

Auto-genotyping results

HIV-1 PR

Consensus length : 297

Rank	Subtype	AC	Score	Match-length	Similarity [%]	Mismatches

Signature(s)

Copyright (C) SmartGene 2001-2023

Sample ID: CQ012022_PGM **Source ID:** CQ012022_PGM **MID:** n.a.

Subtype: F2

1	F1	FJ900268	365	296	90.5	28
2	F1	DQ979024	365	296	90.5	28
3	F1	DQ979025	357	296	90.2	29
4	F1	FJ900266	333	296	89.2	32
5	F1	FJ900267	333	296	89.2	32

HIV-1 RT

Consensus length : 1 620

Rank	Subtype	AC	Score	Match-length	Similarity [%]	Mismatches
1	F2	AJ249236	1 984	1 617	90.6	150
2	F2	MN153483	1 966	1 616	90.3	156
3	F2	KU749420	1 966	1 616	90.3	156
4	CRF46_BF1	MG365771	1 961	1 621	90.4	154
5	F1	FJ771008	1 943	1 620	90.1	160

HIV-1 IN

Consensus length : 864

Rank	Subtype	AC	Score	Match-length	Similarity [%]	Mismatches
1	F2	MN153485	1 302	861	94.1	51
2	D	AF484497	1 302	849	94.3	48
3	A4	AM000054	1 302	861	94.1	51
4	F2	AJ249236	1 287	861	93.8	53
5	F1	FJ900267	1 287	861	93.8	53

Detailed information

**IDNS5 version**	v3.13.0
**Drug resistance algorithm**	ANRS (2022.33/sg/b)
**Pipeline name**	HIV-1 PR+RT_frag+IN
**Pipeline version**	2.4.6_HIV1_v1.6
**Genotyping database version**	HIV1_r23u051
**Noise filter [%]**	0.5
**Interpretation cutoff [%]**	20.0
**Min. read depth [# reads]**	50
**Number of input reads**	752 343
**Number of reads mapped**	680 565
**Mutations HIV-1 PR [(%)]**	3I (100.0), 10V (100.0), 12I (98.0), 13V (100.0), 15V (100.0), 16E (99.7), 20V (98.2), 33F (100.0), 35N (96.9), 36I (100.0), 37N (100.0), 39Q (100.0), 41K (97.9), 45V (100.0), 53L (100.0), 54V (100.0), 57K (94.1), 62V (100.0), 63T (100.0), 74A (100.0), 82F (100.0), 89M (85.8), 90M (100.0)

Signature(s)

Copyright (C) SmartGene 2001-2023

**Sample ID:** CQ012022_PGM	**Source ID:** CQ012022_PGM	**MID:** n.a.
		**Subtype:** F2

**Mutations HIV-1 RT [(%)]**	20R (100.0), 35T (100.0), 39M (100.0), 41L (100.0), 67N (98.4), 69D (98.8), 70R (99.1), 74V (99.2), 75T (100.0), 101Q (98.3), 118I (100.0), 171Y (100.0), 173T (98.3), 174K (94.8), 177E (100.0), 178L (100.0), 181C (100.0), 190A (100.0), 203K (100.0), 207E (100.0), 208Y (100.0), 211A (100.0), 215F (98.4), 219Q (95.6), 223Q (100.0), 228H (99.6), 245Q (100.0), 248D (100.0), 250E (100.0), 277K (100.0), 283I (100.0), 286A (100.0), 291D (100.0), 292I (100.0), 293V (100.0), 297Q (99.4), 324E (100.0), 333D (100.0), 334H (100.0), 356K (89.9), 359S (100.0), 365I (100.0), 376C (100.0), 386I (93.4), 390R (100.0), 399G (100.0), 411L (100.0), 431T (100.0), 449Q (99.5), 451R (100.0), 452E (100.0), 460D (99.5), 466A (100.0), 468S (100.0), 483H (98.9), 491S (100.0), 512K (95.4), 527Q (99.5), 534S (100.0)
**Mutations HIV-1 IN [(%)]**	3E (100.0), 7R (100.0), 10E (100.0), 17N (100.0), 100Y (100.0), 101I (100.0), 112V (100.0), 119P (100.0), 123S (100.0), 125A (100.0), 127K (100.0), 136Q (100.0), 205S (100.0), 218I (99.2), 232D (100.0), 234I (100.0), 255N (100.0), 256E (100.0), 283G (100.0), 288N (94.3)
**Created by**	CH Versailles

Signature(s)

Copyright (C) SmartGene 2001-2023

**Sample ID:** CQ022022_PGM	**Source ID:** CQ022022_PGM	**MID:** n.a.
		**Subtype:** B

Drug resistances

Drug resistance algorithm: ANRS (2022.33/sg/b)

Nucleoside Reverse Transcriptase Inhibitors (NRTI)

Drug	Mutations list	Range	Color	Interpretation
3TC_FTC	**65R (31.7%)**	3		R - Resistance
ABC	**41L (100.0%)**, **65R (31.7%)**, **67N (66.6%)**, **74I (93.4%)**, **215V (96.8%)**	3		R - Resistance
ISL		1		S - Susceptible
TDF_TAF	**41L (100.0%)**, **65R (31.7%)**, **67N (66.6%)**, **69D (67.6%)**, **69N (32.4%)**, **74I (93.4%)**, **215V (96.8%)**	3		R - Resistance
ZDV	**41L (100.0%)**, **67N (66.6%)**, **215V (96.8%)**, **219Q (100.0%)**	3		R - Resistance

Non-Nucleoside Reverse Transcriptase Inhibitors (NNRTI)

Drug	Mutations list	Range	Color	Interpretation
DOR	**181C (100.0%)**	1		S - Susceptible
EFV	**181C (100.0%)**	3		R - Resistance
ETR	**181C (100.0%)**	3		R - Resistance
NVP	**181C (100.0%)**	3		R - Resistance
RPV	**181C (100.0%)**	3		R - Resistance

Protease Inhibitors (PI)

Drug	Mutations list	Range	Color	Interpretation
ATV_RTV	**10V (99.3%)**, **33F (99.3%)**, **46L (100.0%)**, **60E (100.0%)**, **71V (100.0%)**, **84V (100.0%)**	3		R - Resistance
DRV_RTV_BID	**11I (100.0%)**, **33F (99.3%)**, **84V (100.0%)**	2		I - Possible resistance
DRV_RTV_QD	**11I (100.0%)**, **33F (99.3%)**, **84V (100.0%)**	3		R - Resistance
LPVr	**10V (99.3%)**, **24I (100.0%)**, **33F (99.3%)**, **46L (100.0%)**, **54V (99.1%)**, **63P (100.0%)**, **71V (100.0%)**, **82A (100.0%)**, **84V (100.0%)**	3		R - Resistance

Integrase Strand Transfer Inhibitors (INSTI)

Drug	Mutations list	Range	Color	Interpretation
BIC		1		S - Susceptible
CAB		1		S - Susceptible
DTG_BID		1		S - Susceptible
DTG_QD		1		S - Susceptible
EVG		1		S - Susceptible
RAL		1		S - Susceptible

Signature(s)

Copyright (C) SmartGene 2001-2023

SmartGene

NGS - Drug Resistance Report

**Sample ID:** CQ022022_PGM	**Source ID:** CQ022022_PGM	**MID:** n.a.<br>**Subtype:** B

Auto-genotyping results

HIV-1 PR

Consensus length : 297

Rank	Subtype	AC	Score	Match-length	Similarity [%]	Mismatches
1	B	MG572010	414	297	92.6	22
2	B	AF538303	414	297	92.6	22
3	B	AY835758	414	297	92.6	22
4	BD	MH234642	406	297	92.3	23
5	B	KY112061	406	297	92.3	23

HIV-1 RT

Consensus length : 1 617

Rank	Subtype	AC	Score	Match-length	Similarity [%]	Mismatches
1	B	AY037282	2 385	1 614	93.4	106
2	B	D10112	2 383	1 617	93.4	107
3	B	AY835778	2 369	1 614	93.3	108
4	B	AF004394	2 365	1 614	93.3	108
5	B	M38429	2 361	1 614	93.2	109

HIV-1 IN

Consensus length : 867

Rank	Subtype	AC	Score	Match-length	Similarity [%]	Mismatches
1	B	EF637046	1 513	867	97.0	26
2	B	U34604	1 513	867	97.0	26
3	B	ConsB-IN	1 513	867	97.0	26
4	B	JQ316129	1 505	867	96.9	27

**IDNS5 version**	v3.13.0
**Drug resistance algorithm**	ANRS (2022.33/sg/b)
**Pipeline name**	HIV-1 PR+RT_frag+IN
**Pipeline version**	2.6.1_HIV1_v1.6
**Genotyping database version**	HIV1_r23u051
**Noise filter [%]**	0.5
**Interpretation cutoff [%]**	20.0
**Min. read depth [# reads]**	50
**Number of input reads**	337 319
**Number of reads mapped**	289 704
**Mutations HIV-1 PR [(%)]**	3I (100.0), 10V (99.3), 11I (100.0), 13V (100.0), 16A (100.0), 24I (100.0), 33F (99.3), 37N (100.0), 46L (100.0), 54V (99.1), 60E (100.0), 62V (100.0), 63P (100.0), 71V (100.0), 72T (99.5), 73C (100.0), 82A (100.0), 84V (100.0)

Signature(s)

Copyright (C) SmartGene 2001-2023

**Sample ID:** CQ022022_PGM	**Source ID:** CQ022022_PGM	**MID:** n.a.
		**Subtype:** B

5	B	MH234640	1 505	867	96.9	27

Detailed information

**Mutations HIV-1 RT [(%)]**	35M (100.0), 39A (100.0), 41L (100.0), 65R (31.7), 67H (33.4), 67N (66.6), 69D (67.6), 69N (32.4), 70M (28.0), 71G (20.6), 74I (93.4), 75T (100.0), 123N (99.1), 135T (100.0), 138D (100.0), 177E (100.0), 178M (100.0), 181C (100.0), 211T (100.0), 214F (97.2), 215V (96.8), 218E (100.0), 219Q (100.0), 293V (100.0), 297A (100.0), 359S (100.0), 360T (100.0), 376A (100.0), 377V (96.0), 386I (83.3), 390R (99.1), 435A (100.0), 437V (100.0), 460D (99.0), 468S (99.4), 470P (100.0), 491P (100.0), 512K (100.0), 517I (100.0), 519S (100.0), 524E (99.5)
**Mutations HIV-1 IN [(%)]**	10E (100.0), 17N (100.0), 31I (100.0), 45G (97.8), 123S (100.0), 125V (99.3), 127K (100.0), 136N (100.0), 201I (100.0), 232D (100.0)
**Created by**	CH Versailles

Signature(s)

Copyright (C) SmartGene 2001-2023

SmartGene

NGS - Drug Resistance Report

**Sample ID:** CQ032022_PGM	**Source ID:** CQ032022_PGM	**MID:** n.a.
		**Subtype:** B

Drug resistances

Drug resistance algorithm: ANRS (2022.33/sg/b)

Nucleoside Reverse Transcriptase Inhibitors (NRTI)

Drug	Mutations list	Range	Color	Interpretation
3TC_FTC		1		S - Susceptible
ABC	**41L (100.0%)**, **74V (95.6%)**, **215C (83.2%)**	3		R - Resistance
ISL		1		S - Susceptible
TDF_TAF	**41L (100.0%)**, **74V (95.6%)**, **215C (83.2%)**	2		I - Possible resistance
ZDV	**41L (100.0%)**, **215C (83.2%)**, **219E (91.1%)**	3		R - Resistance

Non-Nucleoside Reverse Transcriptase Inhibitors (NNRTI)

Drug	Mutations list	Range	Color	Interpretation
DOR	**100I (100.0%)**, **103N (100.0%)**	3		R - Resistance
EFV	**100I (100.0%)**, **103N (100.0%)**	3		R - Resistance
ETR	**100I (100.0%)**	1		S - Susceptible
NVP	**98S (100.0%)**, **100I (100.0%)**, **103N (100.0%)**	3		R - Resistance
RPV	**100I (100.0%)**, **103N (100.0%)**	3		R - Resistance

Protease Inhibitors (PI)

Drug	Mutations list	Range	Color	Interpretation
ATV_RTV	**10F (99.1%)**, **33F (100.0%)**, **71T (100.0%)**, **84V (100.0%)**, **90M (100.0%)**	3		R - Resistance
DRV_RTV_BID	**32I (100.0%)**, **33F (100.0%)**, **54L (98.8%)**, **84V (100.0%)**, **89V (100.0%)**	3		R - Resistance
DRV_RTV_QD	**32I (100.0%)**, **33F (100.0%)**, **54L (98.8%)**, **84V (100.0%)**, **89V (100.0%)**	3		R - Resistance
LPVr	**10F (99.1%)**, **33F (100.0%)**, **54L (98.8%)**, **63P (100.0%)**, **71T (100.0%)**, **84V (100.0%)**, **90M (100.0%)**	3		R - Resistance

Integrase Strand Transfer Inhibitors (INSTI)

Drug	Mutations list	Range	Color	Interpretation
BIC	**140S (100.0%)**, **148H (97.8%)**	3		R - Resistance
CAB	**140S (100.0%)**, **148H (97.8%)**	3		R - Resistance
DTG_BID	**140S (100.0%)**, **148H (97.8%)**	2		I - Possible resistance
DTG_QD	**140S (100.0%)**, **148H (97.8%)**	3		R - Resistance
EVG	**140S (100.0%)**, **148H (97.8%)**	3		R - Resistance
RAL	**140S (100.0%)**, **148H (97.8%)**	3		R - Resistance

Auto-genotyping results

HIV-1 PR

Consensus length : 297

Signature(s)

Copyright (C) SmartGene 2001-2023

**Sample ID:** CQ032022_PGM	**Source ID:** CQ032022_PGM	**MID:** n.a.
		**Subtype:** B

Rank	Subtype	AC	Score	Match-length	Similarity [%]	Mismatches
1	D	MN650444	408	297	91.9	24
2	B	KY658693	408	297	91.9	24
3	B	EF178358	408	297	91.9	24
4	B	GQ372066	402	297	91.6	25
5	D	MN650466	400	297	91.6	25

HIV-1 RT

Consensus length : 1 158

Rank	Subtype	AC	Score	Match-length	Similarity [%]	Mismatches
1	B	JF689873	1 849	1 158	95.1	57
2	B	M38429	1 845	1 156	95.1	57
3	B	AY835769	1 841	1 158	95.0	58
4	B	JQ316130	1 825	1 158	94.8	60
5	B	KJ140264	1 825	1 158	94.8	60

HIV-1 IN

Consensus length : 867

Rank	Subtype	AC	Score	Match-length	Similarity [%]	Mismatches
1	B	ConsB-IN	1 481	867	96.5	30
2	B	KX505396	1 457	867	96.2	33
3	B	MW405324	1 449	867	96.1	34
4	B	OK181205	1 449	867	96.1	34
5	B	KY514083	1 449	867	96.1	34

Detailed information

**IDNS5 version**	v3.13.0
**Drug resistance algorithm**	ANRS (2022.33/sg/b)
**Pipeline name**	HIV-1 PR+RT_frag+IN
**Pipeline version**	2.4.6_HIV1_v1.6
**Genotyping database version**	HIV1_r23u051
**Noise filter [%]**	0.5
**Interpretation cutoff [%]**	20.0
**Min. read depth [# reads]**	50
**Number of input reads**	573 797
**Number of reads mapped**	490 729
**Mutations HIV-1 PR [(%)]**	3I (100.0), 10F (99.1), 13V (98.0), 32I (100.0), 33F (100.0), 35D (88.9), 36I (100.0), 37N (100.0), 43Q (20.4), 43T (79.5), 54L (98.8), 63P (100.0), 64V (100.0), 71T (100.0), 72V (100.0), 73T (99.8), 84V (100.0), 89V (100.0), 90M (100.0)

Signature(s)

Copyright (C) SmartGene 2001-2023

SmartGene

NGS - Drug Resistance Report

**Sample ID:** CQ032022_PGM	**Source ID:** CQ032022_PGM	**MID:** n.a.
		**Subtype:** B

**Mutations HIV-1 RT [(%)]**	6K (100.0), 41L (100.0), 67G (100.0), 68G (100.0), 74V (95.6), 98S (100.0), 100I (100.0), 103N (100.0), 108I (100.0), 135T (100.0), 177N (99.0), 200I (100.0), 214F (98.1), 215C (83.2), 219E (91.1), 228H (100.0), 272A (100.0), 277K (99.5), 286A (100.0), 297K (97.7), 356K (97.2), 376A (100.0), 379G (21.0)
**Mutations HIV-1 IN [(%)]**	10E (100.0), 20K (100.0), 31I (100.0), 72I (96.6), 101I (96.2), 112M (93.6), 123S (100.0), 124N (100.0), 125V (100.0), 127K (100.0), 140S (100.0), 148H (97.8), 163E (100.0), 207N (99.5), 211T (99.0), 218S (100.0), 232D (100.0), 256E (100.0)
**Created by**	CH Versailles

Signature(s)

Copyright (C) SmartGene 2001-2023

SmartGene

NGS - Drug Resistance Report

**Sample ID:** CQ012023_PGM	**Source ID:** CQ012023_PGM	**MID:** n.a.
		**Subtype:** B

Drug resistances

Drug resistance algorithm: ANRS (2022.33/sg/b)

Nucleoside Reverse Transcriptase Inhibitors (NRTI)

Drug	Mutations list	Range	Color	Interpretation
3TC_FTC	**184V (100.0%)**	3		R - Resistance
ABC	**74I (97.5%)**, **184V (100.0%)**, **215V (99.6%)**	3		R - Resistance
ISL	**184V (100.0%)**	3		R - Resistance
TDF_TAF	**74I (97.5%)**, **215V (99.6%)**	1		S - Susceptible
ZDV	**215V (99.6%)**, **219E (98.3%)**	3		R - Resistance

Non-Nucleoside Reverse Transcriptase Inhibitors (NNRTI)

Drug	Mutations list	Range	Color	Interpretation
DOR	**103N (100.0%)**	1		S - Susceptible
EFV	**103N (100.0%)**	3		R - Resistance
ETR	**138G (100.0%)**	2		I - Possible resistance
NVP	**103N (100.0%)**	3		R - Resistance
RPV	**103N (100.0%)**, **138G (100.0%)**	3		R - Resistance

Protease Inhibitors (PI)

Drug	Mutations list	Range	Color	Interpretation
ATV_RTV	**10V (96.2%)**, **33F (100.0%)**, **60E (100.0%)**, **71V (100.0%)**, **84V (100.0%)**, **90M (100.0%)**	3		R - Resistance
DRV_RTV_BID	**32I (99.4%)**, **33F (100.0%)**, **54L (100.0%)**, **84V (100.0%)**, **89V (100.0%)**	3		R - Resistance
DRV_RTV_QD	**32I (99.4%)**, **33F (100.0%)**, **54L (100.0%)**, **84V (100.0%)**, **89V (100.0%)**	3		R - Resistance
LPVr	**10V (96.2%)**, **33F (100.0%)**, **54L (100.0%)**, **63P (100.0%)**, **71V (100.0%)**, **84V (100.0%)**, **90M (100.0%)**	3		R - Resistance

Integrase Strand Transfer Inhibitors (INSTI)

Drug	Mutations list	Range	Color	Interpretation
BIC		1		S - Susceptible
CAB		1		S - Susceptible
DTG_BID	**97A (100.0%)**	1		S - Susceptible
DTG_QD		1		S - Susceptible
EVG	**97A (100.0%)**, **143R (97.3%)**, **157Q (100.0%)**	3		R - Resistance
RAL	**143R (97.3%)**, **157Q (100.0%)**	3		R - Resistance

Auto-genotyping results

HIV-1 PR

Consensus length : 297

Rank	Subtype	AC	Score	Match-length	Similarity [%]	Mismatches

Signature(s)

Copyright (C) SmartGene 2001-2023

**Sample ID:** CQ012023_PGM	**Source ID:** CQ012023_PGM	**MID:** n.a.	
		**Subtype:** B	

1	B	MF109418	359	297	90.2	29
2	B	AB874132	359	297	90.2	29
3	D	MN694986	351	297	89.9	30
4	B	KY658703	351	297	89.9	30
5	D	AF484480	351	297	89.9	30

HIV-1 RT

**IDNS5 version**	v3.13.0
**Drug resistance algorithm**	ANRS (2022.33/sg/b)
**Pipeline name**	HIV-1 PR+RT_frag+IN
**Pipeline version**	2.7.0_HIV1_v1.6
**Genotyping database version**	HIV1_r23u051
**Noise filter [%]**	0.5
**Interpretation cutoff [%]**	20.0
**Min. read depth [# reads]**	50
**Number of input reads**	185 069
**Number of reads mapped**	125 656
**Mutations HIV-1 PR [(%)]**	3I (100.0), 10V (96.2), 11L (100.0), 13V (100.0), 14R (98.2), 15V (100.0), 20T (100.0), 32I (99. 4), 33F (100.0), 35D (99.1), 36I (100.0), 37N (100.0), 41K (99.5), 54L (100.0), 58E (100. 0), 60E (100.0), 61E (100.0), 62V (100.0), 63P (100.0), 71V (100.0), 73N (100.0), 84V

Signature(s)

Copyright (C) SmartGene 2001-2023

Sample ID: CQ012023_PGM	Source ID: CQ012023_PGM	MID: n.a.
		Subtype: B

(100.0), 89V (100.0), 90M (100.0)

Consensus length : 1 158

Rank	Subtype	AC	Score	Match-length	Similarity [%]	Mismatches
1	B	D10112	1 706	1 155	93.6	74
2	B	AF538303	1 704	1 158	93.5	75
3	B	JF689873	1 696	1 158	93.4	76
4	B	AY835778	1 696	1 158	93.4	76
5	B	AF004394	1 696	1 158	93.4	76

HIV-1 IN

Consensus length : 867

Rank	Subtype	AC	Score	Match-length	Similarity [%]	Mismatches
1	B	M17451	1 441	863	96.1	34
2	B	ConsB-IN	1 413	861	95.7	37
3	B	MW924814	1 382	861	95.2	41
4	B	AY173955	1 378	863	95.1	42
5	B	MW059483	1 374	861	95.1	42

Detailed information

Mutations HIV-1 RT [(%)]
35T (100.0), 62V (99.5), 67G (99.4), 68G (100.0), 74I (97.5), 75T (99.6), 103N (100.0), 135V (100.0), 138G (100.0), 162Y (100.0), 173R (88.4), 174K (94.0), 184V (100.0), 194D (87.6), 207A (100.0), 211K (100.0), 214F (100.0), 215V (99.6), 219E (98.3), 232H (100.0), 238T (100.0), 250N (69.1), 277K (100.0), 293V (99.5), 297A (100.0), 333D (98.9), 335C (100.0), 356K (92.6), 357L (99.1), 360T (99.0), 376A (100.0), 379C (21.9), 379G (27.2)

Mutations HIV-1 IN [(%)]
6E (100.0), 31I (100.0), 72I (98.4), 97A (100.0), 101I (100.0), 111T (100.0), 112V (100.0), 119T (100.0), 123S (100.0), 124N (100.0), 127K (100.0), 142A (97.0), 143R (97.3), 157Q (100.0), 160Q (100.0), 167E (100.0), 218S (100.0), 220M (100.0), 227F (99.4), 230N (99.1), 232D (100.0), 283G (100.0)

Created by
CH Versailles

Signature(s)

Copyright (C) SmartGene 2001-2023

SmartGene

NGS - Drug Resistance Report

Sample ID: CQ022023_PGM **Source ID:** CQ022023_PGM **MID:** n.a.

Subtype: A

Drug resistances

Drug resistance algorithm: ANRS (2022.33/sg/b)

Nucleoside Reverse Transcriptase Inhibitors (NRTI)

Drug	Mutations list	Range	Color	Interpretation
3TC_FTC		1		S - Susceptible
ABC		1		S - Susceptible
ISL		1		S - Susceptible
TDF_TAF		1		S - Susceptible
ZDV		1		S - Susceptible

Non-Nucleoside Reverse Transcriptase Inhibitors (NNRTI)

Drug	Mutations list	Range	Color	Interpretation
DOR	**103N (100.0%)**	1		S - Susceptible
EFV	**103N (100.0%)**	3		R - Resistance
ETR		1		S - Susceptible
NVP	**103N (100.0%)**	3		R - Resistance
RPV	**103N (100.0%)**	1		S - Susceptible

Protease Inhibitors (PI)

Drug	Mutations list	Range	Color	Interpretation
ATV_RTV		1		S - Susceptible
DRV_RTV_BID	**11I (100.0%)**	1		S - Susceptible
DRV_RTV_QD	**11I (100.0%)**	1		S - Susceptible
LPVr		1		S - Susceptible

Integrase Strand Transfer Inhibitors (INSTI)

Drug	Mutations list	Range	Color	Interpretation
BIC	**74I (100.0%)**	1		S - Susceptible
CAB	**74I (100.0%)**	1		S - Susceptible
DTG_BID	**74I (100.0%)**	1		S - Susceptible
DTG_QD	**74I (100.0%)**	1		S - Susceptible
EVG	**74I (100.0%)**	1		S - Susceptible
RAL	**74I (100.0%)**	1		S - Susceptible

Auto-genotyping results

HIV-1 PR

Consensus length : 297

Signature(s)

Copyright (C) SmartGene 2001-2023

NGS - Drug Resistance Report

**Sample ID:** CQ022023_PGM	**Source ID:** CQ022023_PGM	**MID:** n.a.
		**Subtype:** A

Rank	Subtype	AC	Score	Match-length	Similarity [%]	Mismatches
1	A	MH705157	484	296	95.6	13
2	A1	AB485632	476	296	95.3	14
3	CRF02_AG	AY371146	476	296	95.3	14
4	A7	MH078558	468	296	94.9	15
5	CRF01_AE	JX448296	468	296	94.9	15

HIV-1 RT

Consensus length : 1 530

Rank	Subtype	AC	Score	Match-length	Similarity [%]	Mismatches
1	AKU	DQ886038	2 350	1 530	94.1	90
2	A1	M62320	2 092	1 530	92.0	122
3	A3	AY521631	2 068	1 526	91.8	125
4	CRF09_cpx	AY093607	2 068	1 530	91.8	126
5	A1	AB485632	2 064	1 526	91.7	126

HIV-1 IN

Consensus length : 864

Rank	Subtype	AC	Score	Match-length	Similarity [%]	Mismatches
1	A3	AY521629	1 411	864	95.6	38
2	AKU	DQ886038	1 411	864	95.6	38
3	CRF45_cpx	FN392874	1 396	864	95.2	41
4	A6	MN703137	1 394	864	95.2	41
5	CRF45_cpx	FN392877	1 392	860	95.3	40

Detailed information

**IDNS5 version**	v3.13.0
**Drug resistance algorithm**	ANRS (2022.33/sg/b)
**Pipeline name**	HIV-1 PR+RT_frag+IN
**Pipeline version**	2.7.0_HIV1_v1.6
**Genotyping database version**	HIV1_r23u051
**Noise filter [%]**	0.5
**Interpretation cutoff [%]**	20.0
**Min. read depth [# reads]**	50
**Number of input reads**	288 596
**Number of reads mapped**	159 175
**Mutations HIV-1 PR [(%)]**	3I (100.0), 10M (100.0), 11I (100.0), 13V (100.0), 20I (100.0), 35D (99.6), 36I (100.0), 37N (100.0), 41K (100.0), 69K (91.7), 89M (100.0)

Signature(s)

Copyright (C) SmartGene 2001-2023

**Sample ID:** CQ022023_PGM	**Source ID:** CQ022023_PGM	**MID:** n.a.
		**Subtype:** A

**Mutations HIV-1 RT [(%)]**	6K (98.7), 11T (100.0), 28A (100.0), 35T (100.0), 39E (99.4), 103N (100.0), 123S (98.9), 135T (100.0), 173A (84.3), 174K (96.7), 177E (100.0), 196E (100.0), 203A (100.0), 207E (100.0), 211K (100.0), 245Q (100.0), 248D (100.0), 250E (100.0), 277K (100.0), 286A (100.0), 292I (100.0), 293V (98.3), 297R (97.8), 326V (100.0), 335D (100.0), 356K (100.0), 357K (93.6), 359S (98.7), 369A (100.0), 371V (100.0), 375R (45.6), 375V (43.3), 376V (44.1), 376H (46.6), 377Q (45.2), 377L (42.6), 379C (20.6), 379G (20.2), 395R (99.4), 403V (100.0), 432D (93.8), 435A (99.0), 458I (100.0), 460D (100.0), 461K (99.3), 468S (99.2), 471E (100.0), 480H (98.9)
**Mutations HIV-1 IN [(%)]**	10E (100.0), 11D (100.0), 24N (99.3), 42R (100.0), 63I (99.4), 72I (87.5), 74I (100.0), 112I (97.1), 113V (100.0), 123S (100.0), 124N (100.0), 125A (100.0), 127K (100.0), 134D (100.0), 167E (100.0), 201I (100.0), 206S (100.0), 232D (100.0), 234I (100.0), 255T (100.0), 283G (100.0)
**Created by**	CH Versailles

Signature(s)

Copyright (C) SmartGene 2001-2023

SmartGene **NGS - Drug Resistance Report**

**Sample ID:** CQ042023_PGM	**Source ID:** CQ042023_PGM	**MID:** n.a.
		**Subtype:** B

Drug resistances

Drug resistance algorithm: ANRS (2022.33/sg/b)

Nucleoside Reverse Transcriptase Inhibitors (NRTI)

Drug	Mutations list	Range	Color	Interpretation
3TC_FTC		1		S - Susceptible
ABC	**41L (100.0%)**, **215F (85.2%)**	2		I - Possible resistance
ISL		1		S - Susceptible
TDF_TAF	**41L (100.0%)**, **215F (85.2%)**	1		S - Susceptible
ZDV	**41L (100.0%)**, **70R (100.0%)**, **215F (85.2%)**, **219E (93.8%)**	3		R - Resistance

Non-Nucleoside Reverse Transcriptase Inhibitors (NNRTI)

Drug	Mutations list	Range	Color	Interpretation
DOR	**98G (100.0%)**, **181C (100.0%)**	2		I - Possible resistance
EFV	**181C (100.0%)**	3		R - Resistance
ETR	**98G (100.0%)**, **181C (100.0%)**	3		R - Resistance
NVP	**181C (100.0%)**	3		R - Resistance
RPV	**98G (100.0%)**, **181C (100.0%)**	3		R - Resistance

Protease Inhibitors (PI)

Drug	Mutations list	Range	Color	Interpretation
ATV_RTV	**10I (99.3%)**, **71V (100.0%)**, **90M (100.0%)**	3		R - Resistance
DRV_RTV_BID		1		S - Susceptible
DRV_RTV_QD		1		S - Susceptible
LPVr	**10I (99.3%)**, **54V (99.5%)**, **63P (100.0%)**, **71V (100.0%)**, **82A (100.0%)**, **90M (100.0%)**	3		R - Resistance

Integrase Strand Transfer Inhibitors (INSTI)

Drug	Mutations list	Range	Color	Interpretation
BIC		1		S - Susceptible
CAB		1		S - Susceptible
DTG_BID		1		S - Susceptible
DTG_QD		1		S - Susceptible
EVG		1		S - Susceptible
RAL		1		S - Susceptible

Auto-genotyping results

HIV-1 PR

Consensus length : 297

Signature(s)

Copyright (C) SmartGene 2001-2023

Sample ID: CQ042023_PGM **Source ID:** CQ042023_PGM **MID:** n.a.

Subtype: B

Rank	Subtype	AC	Score	Match-length	Similarity [%]	Mismatches
1	B	AB873943	462	293	94.9	15
2	B	FJ469753	462	293	94.9	15
3	B	OK181187	454	293	94.5	16
4	BD	MH234642	454	293	94.5	16
5	B	KY112061	454	293	94.5	16

HIV-1 RT

Consensus length : 1 236

Rank	Subtype	AC	Score	Match-length	Similarity [%]	Mismatches
1	B	FJ469685	1 957	1 239	94.8	62
2	B	AY835778	1 957	1 239	94.8	62
3	B	AF538307	1 957	1 239	94.8	62
4	B	AF538303	1 957	1 239	94.8	62
5	B	K03455	1 957	1 239	94.8	62

HIV-1 IN

Consensus length : 867

Rank	Subtype	AC	Score	Match-length	Similarity [%]	Mismatches
1	B	ConsB-IN	1 497	867	96.8	28
2	B	D10112	1 473	867	96.4	31
3	B	AB564745	1 473	867	96.4	31
4	B	AY173960	1 473	867	96.4	31
5	B	GQ371811	1 467	867	96.3	32

Detailed information

**IDNS5 version**	v3.13.0
**Drug resistance algorithm**	ANRS (2022.33/sg/b)
**Pipeline name**	HIV-1 PR+RT_frag+IN
**Pipeline version**	2.7.0_HIV1_v1.6
**Genotyping database version**	HIV1_r23u051
**Noise filter [%]**	0.5
**Interpretation cutoff [%]**	20.0
**Min. read depth [# reads]**	50
**Number of input reads**	244 915
**Number of reads mapped**	197 317
**Mutations HIV-1 PR [(%)]**	3I (100.0), 10I (99.3), 15V (100.0), 35D (99.6), 36I (100.0), 37N (100.0), 41K (99.4), 54V (99.5), 62V (100.0), 63P (100.0), 71V (100.0), 73S (100.0), 82A (100.0), 90M (100.0)

Signature(s)

Copyright (C) SmartGene 2001-2023

**Sample ID:** CQ042023_PGM	**Source ID:** CQ042023_PGM	**MID:** n.a.
		**Subtype:** B

Mutations HIV-1 RT [(%)]
4T (100.0), 31M (24.8), 36D (100.0), 41L (100.0), 43E (100.0), 67- (76.1), 69G (99.5), 70R (100.0), 98G (100.0), 108I (100.0), 122K (100.0), 123E (100.0), 135T (100.0), 178M (100.0), 181C (100.0), 211K (100.0), 214F (98.8), 215F (85.2), 219E (93.8), 277K (99.1), 293V (100.0), 334H (100.0), 356K (93.7), 359S (99.1), 360T (100.0), 376A (100.0), 379C (21.3), 399K (32.7), 400A (98.9), 401* (30.7)

Mutations HIV-1 IN [(%)]
10E (100.0), 17N (100.0), 28I (99.7), 30A (100.0), 39C (100.0), 112V (100.0), 119P (100.0), 123S (100.0), 124T (100.0), 127K (100.0), 201I (100.0), 232D (100.0), 234I (100.0), 253E (98.3), 286N (99.3)

Created by
CH Versailles

Signature(s)

Copyright (C) SmartGene 2001-2023
