# Supplementary material for: Optimization of HIV Sequencing Method Using Vela Sentosa Library on Miseq Ilumina Platform
Source: Genes (Basel). 2024 Feb 19;15(2):259. doi: 10.3390/genes15020259 (PMC10887851; doi:10.3390/genes15020259)
Supplement: Supplementary file 1 [file genes-15-00259-s001.zip › Supplementary material/File S2.pdf]

SmartGene

NGS - Drug Resistance Report

**Sample ID:** CQ012022_Miseq	**Source ID:** CQ012022_Miseq	**MID:** n.a.
		**Subtype:** F2

Drug resistances

Drug resistance algorithm: ANRS (2022.33/sg/b)

Nucleoside Reverse Transcriptase Inhibitors (NRTI)

Drug	Mutations list	Range	Color	Interpretation
3TC_FTC		1		S - Susceptible
ABC	**41L (100.0%)**, **67N (94.2%)**, **74V (100.0%)**, **215F (99.7%)**	3		R - Resistance
ISL		1		S - Susceptible
TDF_TAF	**41L (100.0%)**, **67N (94.2%)**, **69D (96.6%)**, **74V (100.0%)**, **215F (99.7%)**	3		R - Resistance
ZDV	**41L (100.0%)**, **67N (94.2%)**, **70R (98.0%)**, **215F (99.7%)**, **219Q (100.0%)**	3		R - Resistance

Non-Nucleoside Reverse Transcriptase Inhibitors (NNRTI)

Drug	Mutations list	Range	Color	Interpretation
DOR	**181C (100.0%)**, **190A (100.0%)**	2		I - Possible resistance
EFV	**181C (100.0%)**, **190A (100.0%)**	3		R - Resistance
ETR	**181C (100.0%)**, **190A (100.0%)**	3		R - Resistance
NVP	**181C (100.0%)**, **190A (100.0%)**	3		R - Resistance
RPV	**181C (100.0%)**	3		R - Resistance

Protease Inhibitors (PI)

Drug	Mutations list	Range	Color	Interpretation
ATV_RTV	**10V (100.0%)**, **16E (99.6%)**, **33F (100.0%)**, **90M (100.0%)**	3		R - Resistance
DRV_RTV_BID	**33F (100.0%)**	1		S - Susceptible
DRV_RTV_QD	**33F (100.0%)**	1		S - Susceptible
LPVr	**10V (100.0%)**, **33F (100.0%)**, **53L (99.6%)**, **54V (100.0%)**, **82F (100.0%)**, **90M (100.0%)**	3		R - Resistance

Integrase Strand Transfer Inhibitors (INSTI)

Drug	Mutations list	Range	Color	Interpretation
BIC		1		S - Susceptible
CAB		1		S - Susceptible
DTG_BID		1		S - Susceptible
DTG_QD		1		S - Susceptible
EVG		1		S - Susceptible
RAL		1		S - Susceptible

Auto-genotyping results

HIV-1 PR

Consensus length : 297

Rank	Subtype	AC	Score	Match-length	Similarity [%]	Mismatches

Signature(s)

Copyright (C) SmartGene 2001-2023

**Sample ID:** CQ012022_Miseq	**Source ID:** CQ012022_Miseq	**MID:** n.a.
		**Subtype:** F2

1	F1	FJ900268	365	296	90.5	28
2	F1	DQ979024	365	296	90.5	28
3	F1	DQ979025	357	296	90.2	29
4	F1	FJ900266	333	296	89.2	32
5	F1	FJ900267	333	296	89.2	32

HIV-1 RT

Consensus length : 1 620

Rank	Subtype	AC	Score	Match-length	Similarity [%]	Mismatches
1	F2	AJ249236	1 989	1 617	90.6	150
2	F2	MN153483	1 971	1 616	90.3	156
3	F2	KU749420	1 971	1 616	90.3	156
4	CRF46_BF1	MG365771	1 965	1 621	90.4	154
5	F1	FJ771008	1 955	1 620	90.2	159

HIV-1 IN

Consensus length : 864

Rank	Subtype	AC	Score	Match-length	Similarity [%]	Mismatches
1	F2	MN153485	1 302	861	94.1	51
2	D	AF484497	1 302	849	94.3	48
3	A4	AM000054	1 302	861	94.1	51
4	F2	AJ249236	1 287	861	93.8	53
5	F1	FJ900267	1 287	861	93.8	53

Detailed information

**IDNS5 version**	v3.13.0
**Drug resistance algorithm**	ANRS (2022.33/sg/b)
**Pipeline name**	HIV-1 PR+RT_frag+IN
**Pipeline version**	2.8.0_HIV1_v1.6
**Genotyping database version**	HIV1_r23u051
**Noise filter [%]**	0.5
**Interpretation cutoff [%]**	20.0
**Min. read depth [# reads]**	50
**Number of input reads**	1 005 152
**Number of reads mapped**	558 531
**Mutations HIV-1 PR [(%)]**	3I (100.0), 10V (100.0), 12I (98.5), 13V (100.0), 15V (100.0), 16E (99.6), 20V (98.6), 33F (100.0), 35N (99.7), 36I (100.0), 37N (100.0), 39Q (100.0), 41K (99.6), 45V (100.0), 53L (99.6), 54V (100.0), 57K (99.5), 62V (100.0), 63T (100.0), 74A (100.0), 82F (100.0), 89M (86.4), 90M (100.0)

Signature(s)

Copyright (C) SmartGene 2001-2023

SmartGene **NGS - Drug Resistance Report**

**Sample ID:** CQ012022_Miseq	**Source ID:** CQ012022_Miseq	**MID:** n.a.
		**Subtype:** F2

**Mutations HIV-1 RT [(%)]**	20R (100.0), 35T (100.0), 39M (100.0), 41L (100.0), 67N (94.2), 69D (96.6), 70R (98.0), 74V (100.0), 75T (99.7), 101Q (99.6), 118I (99.6), 171Y (99.6), 173T (99.5), 174K (100.0), 177E (100.0), 178L (99.6), 181C (100.0), 190A (100.0), 203K (99.6), 207E (100.0), 208Y (100.0), 211A (100.0), 215F (99.7), 219Q (100.0), 223Q (100.0), 228H (99.7), 245Q (100.0), 248D (99. 6), 250E (100.0), 277K (99.4), 283I (100.0), 286A (100.0), 291D (99.5), 292I (99.5), 293V (100. 0), 297Q (99.1), 324E (100.0), 333D (99.7), 334H (100.0), 356K (100.0), 359S (99.5), 365I (100.0), 376C (100.0), 386I (60.0), 390R (100.0), 399G (99.7), 411L (100.0), 431T (100.0), 449Q (100.0), 451R (100.0), 452E (100.0), 460D (100.0), 466A (99.6), 468S (100.0), 483H (98.1), 491S (98.3), 512K (100.0), 527Q (99.2), 534S (100.0)
**Mutations HIV-1 IN [(%)]**	3E (100.0), 7R (100.0), 10E (100.0), 17N (100.0), 100Y (99.7), 101I (100.0), 112V (100.0), 119P (100.0), 123S (100.0), 125A (100.0), 127K (100.0), 136Q (100.0), 205S (100.0), 218I (99.3), 232D (100.0), 234I (99.4), 255N (99.6), 256E (100.0), 283G (99.5), 288N (95.2)
**Created by**	CH Versailles

Signature(s)

Copyright (C) SmartGene 2001-2023

NGS - Drug Resistance Report

**Sample ID:** CQ022022_Miseq	**Source ID:** CQ022022_Miseq	**MID:** n.a.
		**Subtype:** B

Drug resistances

Drug resistance algorithm: ANRS (2022.33/sg/b)

Nucleoside Reverse Transcriptase Inhibitors (NRTI)

Drug	Mutations list	Range	Color	Interpretation
3TC_FTC	**65R (42.0%)**	3		R - Resistance
ABC	**41L (100.0%)**, **65R (42.0%)**, **67N (57.8%)**, **74I (99.0%)**, **215V (99.5%)**	3		R - Resistance
ISL		1		S - Susceptible
TDF_TAF	**41L (100.0%)**, **65R (42.0%)**, **67N (57.8%)**, **69D (66.3%)**, **69N (33.1%)**, **74I (99.0%)**, **215V (99.5%)**	3		R - Resistance
ZDV	**41L (100.0%)**, **67N (57.8%)**, **215V (99.5%)**, **219Q (99.4%)**	3		R - Resistance

Non-Nucleoside Reverse Transcriptase Inhibitors (NNRTI)

Drug	Mutations list	Range	Color	Interpretation
DOR	**181C (100.0%)**	1		S - Susceptible
EFV	**181C (100.0%)**	3		R - Resistance
ETR	**181C (100.0%)**	3		R - Resistance
NVP	**181C (100.0%)**	3		R - Resistance
RPV	**181C (100.0%)**	3		R - Resistance

Protease Inhibitors (PI)

Drug	Mutations list	Range	Color	Interpretation
ATV_RTV	**10V (100.0%)**, **33F (100.0%)**, **46L (99.8%)**, **60E (100.0%)**, **71V (100.0%)**, **84V (100.0%)**	3		R - Resistance
DRV_RTV_BID	**11I (100.0%)**, **33F (100.0%)**, **84V (100.0%)**	2		I - Possible resistance
DRV_RTV_QD	**11I (100.0%)**, **33F (100.0%)**, **84V (100.0%)**	3		R - Resistance
LPVr	**10V (100.0%)**, **24I (100.0%)**, **33F (100.0%)**, **46L (99.8%)**, **54V (100.0%)**, **63P (100.0%)**, **71V (100.0%)**, **82A (100.0%)**, **84V (100.0%)**	3		R - Resistance

Integrase Strand Transfer Inhibitors (INSTI)

Drug	Mutations list	Range	Color	Interpretation
BIC		1		S - Susceptible
CAB		1		S - Susceptible
DTG_BID		1		S - Susceptible
DTG_QD		1		S - Susceptible
EVG		1		S - Susceptible
RAL		1		S - Susceptible

Auto-genotyping results

HIV-1 PR

Consensus length : 297

Signature(s)

Copyright (C) SmartGene 2001-2023

**Sample ID:** CQ022022_Miseq	**Source ID:** CQ022022_Miseq	**MID:** n.a.
		**Subtype:** B

Rank	Subtype	AC	Score	Match-length	Similarity [%]	Mismatches
1	B	MG572010	414	297	92.6	22
2	B	AF538303	414	297	92.6	22
3	B	AY835758	414	297	92.6	22
4	BD	MH234642	406	297	92.3	23
5	B	KY112061	406	297	92.3	23

HIV-1 RT

Consensus length : 1 620

Rank	Subtype	AC	Score	Match-length	Similarity [%]	Mismatches
1	B	D10112	2 397	1 620	93.5	106
2	B	AY037282	2 391	1 617	93.4	106
3	B	AY835778	2 383	1 617	93.4	107
4	B	AF004394	2 379	1 617	93.4	107
5	B	M38429	2 367	1 617	93.3	109

HIV-1 IN

Consensus length : 867

Rank	Subtype	AC	Score	Match-length	Similarity [%]	Mismatches
1	B	EF637046	1 513	867	97.0	26
2	B	U34604	1 513	867	97.0	26
3	B	ConsB-IN	1 513	867	97.0	26
4	B	JQ316129	1 505	867	96.9	27
5	B	MH234640	1 505	867	96.9	27

Detailed information

**IDNS5 version**	v3.13.0
**Drug resistance algorithm**	ANRS (2022.33/sg/b)
**Pipeline name**	HIV-1 PR+RT_frag+IN
**Pipeline version**	2.8.0_HIV1_v1.6
**Genotyping database version**	HIV1_r23u051
**Noise filter [%]**	0.5
**Interpretation cutoff [%]**	20.0
**Min. read depth [# reads]**	50
**Number of input reads**	2 074 028
**Number of reads mapped**	1 131 366
**Mutations HIV-1 PR [(%)]**	3I (100.0), 10V (100.0), 11I (100.0), 13V (100.0), 16A (100.0), 24I (100.0), 33F (100.0), 37N (100.0), 46L (99.8), 54V (100.0), 60E (100.0), 62V (100.0), 63P (100.0), 71V (100.0), 72T (100.0), 73C (99.5), 82A (100.0), 84V (100.0)

Signature(s)

Copyright (C) SmartGene 2001-2023

**Sample ID:** CQ022022_Miseq	**Source ID:** CQ022022_Miseq	**MID:** n.a.
		**Subtype:** B

**Mutations HIV-1 RT [(%)]**	35M (98.7), 39A (99.4), 41L (100.0), 65R (42.0), 67H (42.1), 67N (57.8), 69D (66.3), 69N (33.1), 70M (22.4), 74I (99.0), 75T (99.3), 123N (99.4), 135T (99.6), 138D (99.6), 177E (99.6), 178M (99.5), 181C (100.0), 211T (99.5), 214F (99.4), 215V (99.5), 218E (99.5), 219Q (99.4), 293V (99.4), 297A (99.3), 359S (99.1), 360T (99.2), 376A (97.0), 377V (92.3), 386I (32.8), 390R (99.6), 435A (100.0), 437V (100.0), 460D (100.0), 468S (99.3), 470P (100.0), 491P (100.0), 512K (100.0), 517I (100.0), 519S (100.0), 524E (99.4)
**Mutations HIV-1 IN [(%)]**	10E (100.0), 17N (100.0), 31I (100.0), 45G (99.5), 123S (100.0), 125V (99.8), 127K (100.0), 136N (100.0), 201I (100.0), 232D (100.0)
**Created by**	CH Versailles

Signature(s)

Copyright (C) SmartGene 2001-2023

SmartGene

NGS - Drug Resistance Report

Sample ID: CQ032022_Miseq	Source ID: CQ032022_Miseq	MID: n.a.
		Subtype: B

Drug resistances

Drug resistance algorithm: ANRS (2022.33/sg/b)

Nucleoside Reverse Transcriptase Inhibitors (NRTI)

Drug	Mutations list	Range	Color	Interpretation
3TC_FTC		1		S - Susceptible
ABC	**41L (99.7%)**, **74V (99.2%)**, **215C (100.0%)**	3		R - Resistance
ISL		1		S - Susceptible
TDF_TAF	**41L (99.7%)**, **74V (99.2%)**, **215C (100.0%)**	2		I - Possible resistance
ZDV	**41L (99.7%)**, **215C (100.0%)**, **219E (99.2%)**	3		R - Resistance

Non-Nucleoside Reverse Transcriptase Inhibitors (NNRTI)

Drug	Mutations list	Range	Color	Interpretation
DOR	**100I (99.1%)**, **103N (98.9%)**	3		R - Resistance
EFV	**100I (99.1%)**, **103N (98.9%)**	3		R - Resistance
ETR	**100I (99.1%)**	1		S - Susceptible
NVP	**98S (99.1%)**, **100I (99.1%)**, **103N (98.9%)**	3		R - Resistance
RPV	**100I (99.1%)**, **103N (98.9%)**	3		R - Resistance

Protease Inhibitors (PI)

Drug	Mutations list	Range	Color	Interpretation
ATV_RTV	**10F (100.0%)**, **33F (100.0%)**, **71T (99.6%)**, **84V (100.0%)**, **90M (100.0%)**	3		R - Resistance
DRV_RTV_BID	**32I (100.0%)**, **33F (100.0%)**, **54L (100.0%)**, **84V (100.0%)**, **89V (99.9%)**	3		R - Resistance
DRV_RTV_QD	**32I (100.0%)**, **33F (100.0%)**, **54L (100.0%)**, **84V (100.0%)**, **89V (99.9%)**	3		R - Resistance
LPVr	**10F (100.0%)**, **33F (100.0%)**, **54L (100.0%)**, **63P (100.0%)**, **71T (99.6%)**, **84V (100.0%)**, **90M (100.0%)**	3		R - Resistance

Integrase Strand Transfer Inhibitors (INSTI)

Drug	Mutations list	Range	Color	Interpretation
BIC	**140S (100.0%)**, **148H (100.0%)**	3		R - Resistance
CAB	**140S (100.0%)**, **148H (100.0%)**	3		R - Resistance
DTG_BID	**140S (100.0%)**, **148H (100.0%)**	2		I - Possible resistance
DTG_QD	**140S (100.0%)**, **148H (100.0%)**	3		R - Resistance
EVG	**140S (100.0%)**, **148H (100.0%)**	3		R - Resistance
RAL	**140S (100.0%)**, **148H (100.0%)**	3		R - Resistance

Auto-genotyping results

HIV-1 PR

Consensus length : 297

Signature(s)

Copyright (C) SmartGene 2001-2023

**Sample ID:** CQ032022_Miseq	**Source ID:** CQ032022_Miseq	**MID:** n.a.
		**Subtype:** B

Rank	Subtype	AC	Score	Match-length	Similarity [%]	Mismatches
1	D	MN650444	405	297	91.6	25
2	B	KY658693	405	297	91.6	25
3	B	EF178358	405	297	91.6	25
4	B	GQ372066	399	297	91.2	26
5	D	MN650466	397	297	91.2	26

HIV-1 RT

Consensus length : 1 440

Rank	Subtype	AC	Score	Match-length	Similarity [%]	Mismatches
1	B	AY835769	2 312	1 440	95.2	69
2	B	M38429	2 289	1 440	95.0	72
3	B	MH234640	2 265	1 440	94.8	75
4	B	U43096	2 265	1 440	94.8	75
5	B	KJ140264	2 257	1 440	94.7	76

HIV-1 IN

Consensus length : 867

Rank	Subtype	AC	Score	Match-length	Similarity [%]	Mismatches
1	B	ConsB-IN	1 481	867	96.5	30
2	B	KX505396	1 457	867	96.2	33
3	B	MW405324	1 449	867	96.1	34
4	B	OK181205	1 449	867	96.1	34
5	B	KY514083	1 449	867	96.1	34

Detailed information

**IDNS5 version**	v3.13.0
**Drug resistance algorithm**	ANRS (2022.33/sg/b)
**Pipeline name**	HIV-1 PR+RT_frag+IN
**Pipeline version**	2.8.0_HIV1_v1.6
**Genotyping database version**	HIV1_r23u051
**Noise filter [%]**	0.5
**Interpretation cutoff [%]**	20.0
**Min. read depth [# reads]**	50
**Number of input reads**	2 031 110
**Number of reads mapped**	1 003 129
**Mutations HIV-1 PR [(%)]**	3I (100.0), 10F (100.0), 13V (100.0), 32I (100.0), 33F (100.0), 35D (89.9), 36I (100.0), 37N (100.0), 43Q (35.3), 43T (64.3), 45R (32.6), 54L (100.0), 63P (100.0), 64V (99.0), 71T (99.6), 72V (100.0), 73T (99.0), 84V (100.0), 89V (99.9), 90M (100.0)

Signature(s)

Copyright (C) SmartGene 2001-2023

**Sample ID:** CQ032022_Miseq	**Source ID:** CQ032022_Miseq	**MID:** n.a.
		**Subtype:** B

Mutations HIV-1 RT [(%)] 6K (99.5), 39K (37.6), 41L (99.7), 67G (98.7), 68G (99.0), 74V (99.2), 98S (99.1), 100I (99. 1), 103N (98.9), 108I (99.0), 135T (99.4), 177N (98.9), 200I (99.2), 214F (99.4), 215C (100. 0), 219E (99.2), 228H (99.4), 272A (99.1), 277K (98.9), 286A (99.2), 297K (99.1), 356K (99.1), 376A (81.4), 390R (93.4), 458I (98.6), 461T (98.5), 463K (84.4), 468S (100.0)

Mutations HIV-1 IN [(%)] 10E (100.0), 20K (100.0), 31I (100.0), 72I (99.5), 101I (100.0), 112M (100.0), 123S (100.0), 124N (100.0), 125V (100.0), 127K (100.0), 140S (100.0), 148H (100.0), 163E (99.5), 207N (100.0), 211T (99.3), 218S (98.5), 232D (100.0), 256E (100.0)

Created by CH Versailles

Signature(s)

Copyright (C) SmartGene 2001-2023

SmartGene

NGS - Drug Resistance Report

Sample ID: CQ012023_Miseq	Source ID: CQ012023_Miseq	MID: n.a.
		Subtype: B

Drug resistances

Drug resistance algorithm: ANRS (2022.33/sg/b)

Nucleoside Reverse Transcriptase Inhibitors (NRTI)

Drug	Mutations list	Range	Color	Interpretation
3TC_FTC	**184V (99.6%)**	3		R - Resistance
ABC	**74I (100.0%)**, **184V (99.6%)**, **215V (100.0%)**	3		R - Resistance
ISL	**184V (99.6%)**	3		R - Resistance
TDF_TAF	**74I (100.0%)**, **215V (100.0%)**	1		S - Susceptible
ZDV	**215V (100.0%)**, **219E (99.6%)**	3		R - Resistance

Non-Nucleoside Reverse Transcriptase Inhibitors (NNRTI)

Drug	Mutations list	Range	Color	Interpretation
DOR	**103N (99.3%)**	1		S - Susceptible
EFV	**103N (99.3%)**	3		R - Resistance
ETR	**138G (99.6%)**	2		I - Possible resistance
NVP	**103N (99.3%)**	3		R - Resistance
RPV	**103N (99.3%)**, **138G (99.6%)**	3		R - Resistance

Protease Inhibitors (PI)

Drug	Mutations list	Range	Color	Interpretation
ATV_RTV	**10V (99.2%)**, **33F (99.2%)**, **60E (100.0%)**, **71V (100.0%)**, **84V (100.0%)**, **90M (100.0%)**	3		R - Resistance
DRV_RTV_BID	**32I (100.0%)**, **33F (99.2%)**, **54L (99.3%)**, **84V (100.0%)**, **89V (99.8%)**	3		R - Resistance
DRV_RTV_QD	**32I (100.0%)**, **33F (99.2%)**, **54L (99.3%)**, **84V (100.0%)**, **89V (99.8%)**	3		R - Resistance
LPVr	**10V (99.2%)**, **33F (99.2%)**, **54L (99.3%)**, **63P (100.0%)**, **71V (100.0%)**, **84V (100.0%)**, **90M (100.0%)**	3		R - Resistance

Integrase Strand Transfer Inhibitors (INSTI)

Drug	Mutations list	Range	Color	Interpretation
BIC		1		S - Susceptible
CAB		1		S - Susceptible
DTG_BID	**97A (99.3%)**	1		S - Susceptible
DTG_QD		1		S - Susceptible
EVG	**97A (99.3%)**, **143R (96.6%)**, **157Q (100.0%)**	3		R - Resistance
RAL	**143R (96.6%)**, **157Q (100.0%)**	3		R - Resistance

Auto-genotyping results

HIV-1 PR

Consensus length : 297

Rank	Subtype	AC	Score	Match-length	Similarity [%]	Mismatches

Signature(s)

Copyright (C) SmartGene 2001-2023

Sample ID: CQ012023_Miseq **Source ID:** CQ012023_Miseq **MID:** n.a.

Subtype: B

1	B	AB874132	367	297	90.6	28
2	B	KY658703	359	297	90.2	29
3	D	AF484480	359	297	90.2	29
4	B	EU839606	359	297	90.2	29
5	B	MW059247	351	297	89.9	30

HIV-1 RT

**IDNS5 version**	v3.13.0
**Drug resistance algorithm**	ANRS (2022.33/sg/b)
**Pipeline name**	HIV-1 PR+RT_frag+IN
**Pipeline version**	2.8.0_HIV1_v1.6
**Genotyping database version**	HIV1_r23u051
**Noise filter [%]**	0.5
**Interpretation cutoff [%]**	20.0
**Min. read depth [# reads]**	50
**Number of input reads**	2 032 146
**Number of reads mapped**	1 059 312
**Mutations HIV-1 PR [(%)]**	3I (100.0), 10V (99.2), 11L (100.0), 13V (100.0), 14R (100.0), 15V (100.0), 20T (100.0), 32I (100.0), 33F (99.2), 35D (97.7), 36I (100.0), 37N (100.0), 41K (100.0), 54L (99.3), 58E (98.5), 60E (100.0), 61E (100.0), 62V (100.0), 63P (100.0), 71V (100.0), 73N (99.4), 84V

Signature(s)

Copyright (C) SmartGene 2001-2023

Sample ID: CQ012023_Miseq	Source ID: CQ012023_Miseq	MID: n.a.
		Subtype: B

(100.0), 89V (99.8), 90M (100.0)

Consensus length : 1 476

Rank	Subtype	AC	Score	Match-length	Similarity [%]	Mismatches
1	B	AF538303	2 114	1 476	93.0	103
2	B	M17451	2 106	1 476	93.0	104
3	B	JF689873	2 098	1 476	92.9	105
4	B	AF004394	2 098	1 476	92.9	105
5	B	AY835769	2 074	1 476	92.7	108

HIV-1 IN

Consensus length : 867

Rank	Subtype	AC	Score	Match-length	Similarity [%]	Mismatches
1	B	M17451	1 441	863	96.1	34
2	B	ConsB-IN	1 413	861	95.7	37
3	B	MW924814	1 382	861	95.2	41
4	B	AY173955	1 378	863	95.1	42
5	B	MW059483	1 374	861	95.1	42

Detailed information

Mutations HIV-1 RT [(%)]
35T (99.1), 62V (98.6), 67G (100.0), 68G (99.6), 74I (100.0), 75T (99.8), 103N (99.3), 135V (99.1), 138G (99.6), 162Y (99.8), 173R (97.0), 174K (99.4), 184V (99.6), 194D (94.6), 207A (99.8), 211K (99.4), 214F (100.0), 215V (100.0), 219E (99.6), 232H (99.6), 238T (99.5), 250N (95.7), 277K (99.5), 293V (97.1), 297A (99.6), 333D (99.3), 335C (99.5), 356K (99.4), 357L (99.2), 360T (98.9), 376A (99.0), 379C (21.0), 390R (98.0), 399D (98.8), 405H (98.7), 435I (98.9), 460D (98.8), 468S (100.0), 470N (99.2), 491S (100.0)

Mutations HIV-1 IN [(%)]
6E (100.0), 31I (100.0), 72I (100.0), 97A (99.3), 101I (100.0), 111T (99.6), 112V (100.0), 119T (100.0), 123S (100.0), 124N (100.0), 127K (100.0), 142A (94.9), 143R (96.6), 157Q (100.0), 160Q (99.5), 167E (100.0), 218S (98.2), 220M (100.0), 227F (100.0), 230N (99.6), 232D (100.0), 283G (100.0)

Created by
CH Versailles

Signature(s)

Copyright (C) SmartGene 2001-2023

**Sample ID:** CQ022023_Miseq	**Source ID:** CQ022023_Miseq	**MID:** n.a.
		**Subtype:** A1

Drug resistances

Drug resistance algorithm: ANRS (2022.33/sg/b)

Nucleoside Reverse Transcriptase Inhibitors (NRTI)

Drug	Mutations list	Range	Color	Interpretation
3TC_FTC		1		S - Susceptible
ABC		1		S - Susceptible
ISL		1		S - Susceptible
TDF_TAF		1		S - Susceptible
ZDV		1		S - Susceptible

Non-Nucleoside Reverse Transcriptase Inhibitors (NNRTI)

Drug	Mutations list	Range	Color	Interpretation
DOR	**103N (99.3%)**	1		S - Susceptible
EFV	**103N (99.3%)**	3		R - Resistance
ETR		1		S - Susceptible
NVP	**103N (99.3%)**	3		R - Resistance
RPV	**103N (99.3%)**	1		S - Susceptible

Protease Inhibitors (PI)

Drug	Mutations list	Range	Color	Interpretation
ATV_RTV		1		S - Susceptible
DRV_RTV_BID	**11I (99.3%)**	1		S - Susceptible
DRV_RTV_QD	**11I (99.3%)**	1		S - Susceptible
LPVr		1		S - Susceptible

Integrase Strand Transfer Inhibitors (INSTI)

Drug	Mutations list	Range	Color	Interpretation
BIC	**74I (100.0%)**	1		S - Susceptible
CAB	**74I (100.0%)**	1		S - Susceptible
DTG_BID	**74I (100.0%)**	1		S - Susceptible
DTG_QD	**74I (100.0%)**	1		S - Susceptible
EVG	**74I (100.0%)**	1		S - Susceptible
RAL	**74I (100.0%)**	1		S - Susceptible

Auto-genotyping results

HIV-1 PR

Consensus length : 297

Signature(s)

Copyright (C) SmartGene 2001-2023

Sample ID: CQ022023_Miseq **Source ID:** CQ022023_Miseq **MID:** n.a.

Subtype: A1

Rank	Subtype	AC	Score	Match-length	Similarity [%]	Mismatches
1	A	MH705157	484	296	95.6	13
2	A1	AB485632	476	296	95.3	14
3	CRF02_AG	AY371146	476	296	95.3	14
4	A7	MH078558	468	296	94.9	15
5	CRF01_AE	JX448296	468	296	94.9	15

HIV-1 RT

Consensus length : 1 617

Rank	Subtype	AC	Score	Match-length	Similarity [%]	Mismatches
1	A1	M62320	2 190	1 613	91.9	130
2	A1	AB485632	2 178	1 617	91.7	134
3	CRF09_cpx	AY093607	2 174	1 617	91.7	134
4	A1	MH705153	2 166	1 617	91.7	134
5	A3	AY521631	2 150	1 617	91.5	137

HIV-1 IN

Consensus length : 864

Rank	Subtype	AC	Score	Match-length	Similarity [%]	Mismatches
1	A3	AY521629	1 411	864	95.6	38
2	AKU	DQ886038	1 411	864	95.6	38
3	CRF45_cpx	FN392874	1 396	864	95.2	41
4	A6	MN703137	1 394	864	95.2	41
5	CRF45_cpx	FN392877	1 392	860	95.3	40

Detailed information

**IDNS5 version**	v3.13.0
**Drug resistance algorithm**	ANRS (2022.33/sg/b)
**Pipeline name**	HIV-1 PR+RT_frag+IN
**Pipeline version**	2.8.0_HIV1_v1.6
**Genotyping database version**	HIV1_r23u051
**Noise filter [%]**	0.5
**Interpretation cutoff [%]**	20.0
**Min. read depth [# reads]**	50
**Number of input reads**	1 675 712
**Number of reads mapped**	630 450
**Mutations HIV-1 PR [(%)]**	3I (100.0), 10M (100.0), 11I (99.3), 13V (100.0), 20I (100.0), 35D (99.6), 36I (100.0), 37N (100.0), 41K (99.5), 69K (100.0), 89M (100.0)

Signature(s)

Copyright (C) SmartGene 2001-2023

**Sample ID:** CQ022023_Miseq	**Source ID:** CQ022023_Miseq	**MID:** n.a.
		**Subtype:** A1

**Mutations HIV-1 RT [(%)]**	6K (100.0), 11T (99.6), 28A (99.2), 35T (100.0), 39E (99.3), 103N (99.3), 123S (98.0), 135T (99.5), 173A (99.2), 174K (98.6), 177E (99.5), 196E (99.2), 203A (99.4), 207E (100.0), 211K (99.2), 245Q (99.4), 248D (99.3), 250E (99.5), 277K (99.1), 286A (99.3), 292I (99.1), 293V (99.3), 297R (98.5), 326V (98.4), 335D (99.1), 356K (99.1), 357K (99.4), 359S (98.6), 369A (98.6), 371V (97.9), 375R (39.6), 375V (54.6), 376V (52.4), 376H (41.2), 377Q (40.4), 377L (50.6), 395R (99.3), 403V (99.5), 432D (99.3), 435A (100.0), 458I (98.0), 460D (98.8), 461K (99.4), 468S (97.6), 471E (100.0), 480H (99.4), 512K (98.1), 519S (100.0), 524K (97.6), 527E (83.5), 530R (100.0), 534S (99.1)
**Mutations HIV-1 IN [(%)]**	10E (100.0), 11D (100.0), 24N (100.0), 42R (99.6), 63I (100.0), 72I (100.0), 74I (100.0), 112I (100.0), 113V (99.7), 123S (100.0), 124N (100.0), 125A (100.0), 127K (100.0), 134D (99.7), 167E (100.0), 201I (100.0), 206S (99.6), 232D (100.0), 234I (100.0), 255T (100.0), 283G (100.0)
**Created by**	CH Versailles

Signature(s)

Copyright (C) SmartGene 2001-2023

SmartGene **NGS - Drug Resistance Report**

**Sample ID:** CQ042023_Miseq	**Source ID:** CQ042023_Miseq	**MID:** n.a.
		**Subtype:** B

Drug resistances

Drug resistance algorithm: ANRS (2022.33/sg/b)

Nucleoside Reverse Transcriptase Inhibitors (NRTI)

Drug	Mutations list	Range	Color	Interpretation
3TC_FTC		1		S - Susceptible
ABC	41L (100.0%), 215F (100.0%)	2		I - Possible resistance
ISL		1		S - Susceptible
TDF_TAF	**41L (100.0%)**, **215F (100.0%)**	1		S - Susceptible
ZDV	41L (100.0%), 70R (93.9%), 215F (100.0%), 219E (99.5%)	3		R - Resistance

Non-Nucleoside Reverse Transcriptase Inhibitors (NNRTI)

Drug	Mutations list	Range	Color	Interpretation
DOR	98G (98.1%), 181C (100.0%)	2		I - Possible resistance
EFV	181C (100.0%)	3		R - Resistance
ETR	**98G (98.1%)**, 181C (100.0%)	3		R - Resistance
NVP	181C (100.0%)	3		R - Resistance
RPV	98G (98.1%), 181C (100.0%)	3		R - Resistance

Protease Inhibitors (PI)

Drug	Mutations list	Range	Color	Interpretation
ATV_RTV	10I (100.0%), 71V (100.0%), 90M (100.0%)	3		R - Resistance
DRV_RTV_BID		1		S - Susceptible
DRV_RTV_QD		1		S - Susceptible
LPVr	10I (100.0%), 54V (100.0%), 63P (100.0%), 71V (100.0%), 82A (100.0%), 90M (100.0%)	3		R - Resistance

Integrase Strand Transfer Inhibitors (INSTI)

Drug	Mutations list	Range	Color	Interpretation
BIC		1		S - Susceptible
CAB		1		S - Susceptible
DTG_BID		1		S - Susceptible
DTG_QD		1		S - Susceptible
EVG		1		S - Susceptible
RAL		1		S - Susceptible

Auto-genotyping results

HIV-1 PR

Consensus length : 297

Signature(s)

Copyright (C) SmartGene 2001-2023

Sample ID: CQ042023_Miseq **Source ID:** CQ042023_Miseq **MID:** n.a.

Subtype: B

Rank	Subtype	AC	Score	Match-length	Similarity [%]	Mismatches
1	B	AB873943	462	293	94.9	15
2	B	FJ469753	462	293	94.9	15
3	B	OK181187	454	293	94.5	16
4	BD	MH234642	454	293	94.5	16
5	B	KY112061	454	293	94.5	16

HIV-1 RT

Consensus length : 1 410

Rank	Subtype	AC	Score	Match-length	Similarity [%]	Mismatches
1	B	AF538307	2 242	1 413	95.0	67
2	B	AY835778	2 236	1 394	95.3	63
3	B	K03455	2 236	1 394	95.3	63
4	B	AF538303	2 234	1 413	95.0	68
5	B	FJ469685	2 226	1 413	94.9	69

HIV-1 IN

Consensus length : 867

Rank	Subtype	AC	Score	Match-length	Similarity [%]	Mismatches
1	B	ConsB-IN	1 497	867	96.8	28
2	B	D10112	1 473	867	96.4	31
3	B	AB564745	1 473	867	96.4	31
4	B	AY173960	1 473	867	96.4	31
5	B	GQ371811	1 467	867	96.3	32

Detailed information

**IDNS5 version**	v3.13.0
**Drug resistance algorithm**	ANRS (2022.33/sg/b)
**Pipeline name**	HIV-1 PR+RT_frag+IN
**Pipeline version**	2.8.0_HIV1_v1.6
**Genotyping database version**	HIV1_r23u051
**Noise filter [%]**	0.5
**Interpretation cutoff [%]**	20.0
**Min. read depth [# reads]**	50
**Number of input reads**	642 162
**Number of reads mapped**	331 310
**Mutations HIV-1 PR [(%)]**	3I (100.0), 10I (100.0), 15V (100.0), 35D (98.6), 36I (100.0), 37N (100.0), 41K (100.0), 54V (100.0), 62V (100.0), 63P (100.0), 71V (100.0), 73S (98.2), 82A (100.0), 90M (100.0)

Signature(s)

Copyright (C) SmartGene 2001-2023

SmartGene

NGS - Drug Resistance Report

**Sample ID:** CQ042023_Miseq	**Source ID:** CQ042023_Miseq	**MID:** n.a.
		**Subtype:** B

Mutations HIV-1 RT [(%)] 4T (100.0), 36D (99.6), 41L (100.0), 43E (97.2), 67- (86.7), 69G (99.9), 70R (93.9), 98G (98. 1), 108I (99.5), 122K (99.6), 123E (100.0), 135T (100.0), 178M (99.5), 181C (100.0),
211K (99.4), 214F (98.6), 215F (100.0), 219E (99.5), 277K (99.4), 293V (100.0), 334H (99. 7), 356K (98.7), 359S (99.3), 360T (99.5), 376A (99.1), 400A (100.0), 460D (98.4), 466I (100.0), 467I (100.0), 468S (98.2), 470N (100.0)

Mutations HIV-1 IN [(%)] 10E (100.0), 17N (100.0), 28I (99.8), 30A (100.0), 39C (100.0), 112V (99.9), 119P (100.0), 123S (100.0), 124T (99.2), 127K (100.0), 201I (100.0), 232D (100.0), 234I (100.0), 253E (100.0), 286N (99.3)

Created by CH Versailles

Signature(s)

Copyright (C) SmartGene 2001-2023
